# Supplementary material for: Smartphone use behavior and quality of life: What is the role of awareness?
Source: PLoS One. 2022 Mar 11;17(3):e0260637. doi: 10.1371/journal.pone.0260637 (PMC8916658; doi:10.1371/journal.pone.0260637)
Supplement: S1 File — (DOCX) [file pone.0260637.s001.docx]

# S1 File

1. **SEM models**

MODEL 1 - QOL to Usability

'competence =~ y2 + y12 + y15 + y16 + y17 + y18
                functioning =~ y3 +y4+y5+y6+y9+y10+y11+y13+y14+ y21
                positive_feeling =~ y1 + y7 + y8 + y19 + y20 + y22  
                unaware =~ x4+x3+x5+x6+x12+x11+x10
                aware =~ x1+x2+x7+x8+x9
                y13 ~~ y14
               y4 ~~ y3
               y12 ~~ y15
               competence ~ unaware + aware
               functioning ~  unaware + aware
               positive_feeling ~  unaware + aware'

MODEL 2: Usability to QOL

full.model2 <- 'competence =~ y2 + y12 + y15 + y16 + y17 + y18
                functioning =~ y3 +y4+y5+y6+y9+y10+y11+y13+y14+ y21
                positive_feeling =~ y1 + y7 + y8 + y19 + y20 + y22
                unaware =~ x4+x3+x5+x6+x12+x11+x10
                aware =~ x1+x2+x7+x8+x9
                y13 ~~ y14
               y4 ~~ y3
               y12 ~~ y15               
               unaware ~ competence + functioning + positive_feeling
               aware ~ competence + functioning + positive_feeling'

1. **CFA Analysis**

| **S1 Table. CFA analysis.** |  | | |  | |  | |  | |  |
| --- | --- | --- | --- | --- | --- | --- | --- | --- | --- | --- |
| **Latent Variables: CFI** | **Estimate** | | | **Std.Err** | | **z-value** | | **P(>\|z\|)** | |  |
| **Competence** |  | | |  | |  | |  | |  |
| y2 | 1 | | |  | |  | |  | |  |
| y12 | 1.04 | | | 0.168 | | 6.196 | | 0 | |  |
| y15 | 1.397 | | | 0.182 | | 7.686 | | 0 | |  |
| y16 | 1.61 | | | 0.183 | | 8.79 | | 0 | |  |
| y17 | 1.724 | | | 0.189 | | 9.139 | | 0 | |  |
| y18 | 1.548 | | | 0.175 | | 8.823 | | 0 | |  |
| **Functioning** |  | | |  | |  | |  | |  |
| y3 | 1 | | |  | |  | |  | |  |
| y4 | 0.934 | | | 0.186 | | 5.023 | | 0 | |  |
| y5 | 1.715 | | | 0.261 | | 6.579 | | 0 | |  |
| y6 | 1.525 | | | 0.246 | | 6.211 | | 0 | |  |
| y9 | 1.475 | | | 0.241 | | 6.122 | | 0 | |  |
| y10 | 1.853 | | | 0.278 | | 6.675 | | 0 | |  |
| y11 | 1.551 | | | 0.238 | | 6.505 | | 0 | |  |
| y13 | 1.481 | | | 0.232 | | 6.382 | | 0 | |  |
| y14 | 1.541 | | | 0.237 | | 6.49 | | 0 | |  |
| y21 | 1.584 | | | 0.245 | | 6.467 | | 0 | |  |
| **Positive_feeling** |  | | |  | |  | |  | |  |
| y1 | 1 | | |  | |  | |  | |  |
| y7 | 1.389 | | | 0.216 | | 6.418 | | 0 | |  |
| y8 | 0.958 | | | 0.143 | | 6.71 | | 0 | |  |
| y19 | 1.38 | | | 0.171 | | 8.061 | | 0 | |  |
| y20 | 1.49 | | | 0.171 | | 8.709 | | 0 | |  |
| y22 | 1.661 | | | 0.188 | | 8.852 | | 0 | |  |
| **Unaware** |  | | |  | |  | |  | |  |
| x4 | 1 | | |  | |  | |  | |  |
| x3 | 0.849 | | | 0.123 | | 6.92 | | 0 | |  |
| x5 | 0.886 | | | 0.101 | | 8.776 | | 0 | |  |
| x6 | 0.607 | | | 0.093 | | 6.493 | | 0 | |  |
| x12 | 0.778 | | | 0.1 | | 7.791 | | 0 | |  |
| x11 | 0.897 | | | 0.127 | | 7.087 | | 0 | |  |
| x10 | 0.836 | | | 0.138 | | 6.035 | | 0 | |  |
| **Aware** |  | | |  | |  | |  | |  |
| x1 | 1 | | |  | |  | |  | |  |
| x2 | 0.748 | | | 0.176 | | 4.242 | | 0 | |  |
| x7 | 1.29 | | | 0.306 | | 4.216 | | 0 | |  |
| x8 | 0.995 | | | 0.235 | | 4.242 | | 0 | |  |
| x9 | 0.752 | | | 0.178 | | 4.22 | | 0 | |  |
| **S2 Table. Fit measures** | | |  |  |  |  |  |  |  |  |
| **User Model versus Baseline Model:** | **Value** | |  |  |  |  |  |  |  |  |
| Comparative Fit Index (CFI) | 0.913 | |  |  |  |  |  |  |  |  |
| Tucker-Lewis Index (TLI) | 0.928 | |  |  |  |  |  |  |  |  |
| RMSEA | 0.049 | |  |  |  |  |  |  |  |  |
| SRMR | 0.061 | |  |  |  |  |  |  |  |  |
| **S3 Table. Regression** | | | | | | | | |  | |
| **Regressions:** | | **Estimate** | | | **Std.Err** | | **z-value** | | **P(>\|z\|)** | |
| Competence ~ Unaware | | -0.398 | | | 0.128 | | -3.119 | | 0.002 | |
| Competence ~ Aware | | 0.319 | | | 0.195 | | 1.631 | | 0.103 | |
| Functioning ~ Unaware | | -0.333 | | | 0.102 | | -3.275 | | 0.001 | |
| Functioning ~ Aware | | 0.223 | | | 0.145 | | 1.543 | | 0.123 | |
| Positive_feeling ~ Unaware | | -0.408 | | | 0.123 | | -3.332 | | 0.001 | |
| Positive_feeling ~ Aware | | 0.5 | | | 0.203 | | 2.466 | | 0.014 | |

1. **Reliability estimations**

We tested the reliability by using both the Cronbach's Alpha and the McDonald's Omega estimators. The QOL Cronbach's Alpha of the *Competence*, *Functioning* and *Positive Feelings* latent variables were 0.86, 0.92, 0.82 respectively (see Table SI-4 below). For the Aware component is slightly lower than 0.6, which is low, but nevertheless, it is considered as not unacceptable (eg, George & Mallery, 2003). Also, as can be seen from the regression coefficient in Table SI-3, the Aware and the Unaware coefficients are in inverse directions for all 3 usability variables, i.e. (-0.398, 0.319), (-0.333, 0.223), (-0.408, 0.5). Thus, these factors, although possibly not clustered around a single clear latent variable named Aware usability, are different from the Unaware usability variables.

| **S4 Table. Reliability estimates - Cronbach Alpha & McDonald`s Omega** | | | | | |
| --- | --- | --- | --- | --- | --- |
| **Reliability by Cronbach Alpha and McDonald`s Omega** | **Competence** | **Functioning** | **Positive feeling** | **Unaware** | **Aware** |
| Cronbach Alpha | 0.859 | 0.902 | 0.8217 | 0.761 | 0.592 |
| McDonald's Omega | 0.862 | 0.904 | 0.823 | 0.774 | 0.594 |

Also, we tried to remove from the Aware latent variable X1, X2, X8, X9, but this did not result in a better reliability for the Aware construct. Finally, we performed an interclass correlation coefficient analysis, (see Table SI-5 below). One can see that the Cronbach Alpha for the Aware construct is within the 95% confidence intervals.

| **S5 Table. Interclass correlation coefficient** | | | | | |
| --- | --- | --- | --- | --- | --- |
|  |  | **95% Confidence interval** | |  |  |
|  | **Interclass Correlation** | **Lower Bound** | **Upper Bound** | **Value** | **Significance** |
| Single Measure | 0.225 | 0.166 | 0.292 | 2.45 | 0 |
| Average Measure | 0.592 | 0.498 | 0.673 | 2.45 | 0 |

1. **Sample size**

The sample size was based on a pre-experiment analysis of the first 50 respondents, in which we estimated the normalized QOL standard deviation SD=0.22. Based on this estimate, the sample size was defined (Z-score=1.96, 95% confidence level, 5% precision), to be $\frac{{1.96}^{2}*0.22*(1-0.22)}{0.0025}=263$ respondents. As the data collection from the questionnaire progressed, we updated the standard deviation estimations to the real values of 0.194. These corresponded with a sample size of 234 individuals. Following this stage, 19 questionnaires were excluded due to poor data quality, resulting in 215 full usable questionnaires. This sample size fits a precision level of 0.052, which is slightly higher that the desired 0.05 precision level but based on higher quality and more reliable questionnaires. Furthermore, the SD of the Aware/Unaware latent variables was slightly smaller (0.19/0.17) respectively, supporting our decision.

1. **QOL questionnaire.**

These questions, copied from the original QOL study [36], measure the levels of QOL through the Positive feelings, Competence and Functioning subfactors.

**S6 Table. QOL Questionnaire**

| Question | There is a lot | To some extent | Not so much | None at all |
| --- | --- | --- | --- | --- |
| A general sense of good physical health |  |  |  |  |
| Pain |  |  |  |  |
| Work (outside the home or in the home, including caring for the household) |  |  |  |  |
| Activity (outside the home or inside the home) |  |  |  |  |
| Strength and ability to perform the activities of eating, sleeping, etc. |  |  |  |  |
| Strength and ability to perform activities in the family (as a partner, parent, sibling, son/daughter) |  |  |  |  |
| Intimate relations with your partner |  |  |  |  |
| Relations with your friends, acquaintances, relatives |  |  |  |  |
| Taking care of yourself and your external appearance |  |  |  |  |
| Strength and ability to cope with the tasks of your everyday life |  |  |  |  |
| Independence in functioning and activities in your daily life |  |  |  |  |
| Not experiencing a sense of a lack of control over situations, feeling that you cannot determine anything that happens |  |  |  |  |
| Ability to concentrate on the task you are performing |  |  |  |  |
| Ability to think, solve problems |  |  |  |  |
| Sense of uncertainty, lack of clarity |  |  |  |  |
| Anxiety, fear |  |  |  |  |
| Depression, sadness |  |  |  |  |
| Tension, restlessness |  |  |  |  |
| Hope |  |  |  |  |
| Pleasure |  |  |  |  |
| Motivation to make an effort and continue doing things |  |  |  |  |
| Satisfaction with life in general |  |  |  |  |

The entire questionnaire as well as the Mechanical Turk experiment can be found in <https://bit.ly/2YWiR7z>
