# Supplementary material for: Smartphone use behavior and quality of life: What is the role of awareness?
Source: PLoS One. 2022 Mar 11;17(3):e0260637. doi: 10.1371/journal.pone.0260637 (PMC8916658; doi:10.1371/journal.pone.0260637)
Supplement: Attachment — Submitted filename: PONE-D-21-20673.docx [file pone.0260637.s002.docx]

PLOS ONE

Smartphone Use Behavior and Quality of Life: What Is the Role of Awareness?

--Manuscript Draft--

| Manuscript Number: | PONE-D-21-20673 |
| --- | --- |
| Article Type: | Research Article |
| Full Title: | Smartphone Use Behavior and Quality of Life: What Is the Role of Awareness? |
| Short Title: | Smartphone Use Behavior and Quality of Life. |
| Corresponding Author: | Alon Sela, Ph.D.  Ariel University  Ariel, Israel ISRAEL |
| Keywords: | Smartphone addiction; quality of life; data science; split attention; cognitive load; digital living |
| Abstract: | How does smartphone use behavior affect quality of life factors? The following work suggests new insights into smartphone use behavior, mainly regarding two  contradicting smartphone modes of use that affect quality of life in opposite ways. The Aware smartphone mode of use reflects an active lifestyle, while the Unaware mode of use reflects the use of the smartphone in conjunction with other activities. Using data from 215 individuals who reported their quality of life and smartphone use habits, we show that high levels of smartphone use in the Unaware mode of use have a significant negative effect on the quality of life. However, the results show a mild positive effect when the individual uses the smartphone in an aware mode of use. We identify three latent factors within the quality of life construct and measure the effect of the different smartphone modes of use on these quality of life factors. We find that (i) The functioning latent factor, which is an individual’s ability to function well in his or her daily life, is not affected by smartphone use behavior. In contrast, (ii) the competence latent factor, which is a lack of negative emotions or pain, and (iii) the positive feelings latent factor both show a clear effect with the smartphone Unaware mode of use. This implies that the unaware use of smartphones, which is its use in conjunction with other activities or late at night, can be related to lower levels of quality of life. Since smartphones currently serve as an interface between the self and the cyber space, as well as an interface between the self and other individuals online, these results need to be considered for social wellbeing in relation to digital human behavior, smartphone addiction and a healthy mode of use. |
| Order of Authors: | Alon Sela, Ph.D. |
|  | Noam Rozenboim |
|  | Hila Chalutz Ben-Gal, Ph.D. |
| Additional Information: |  |
| Question | Response |
| Financial Disclosure  Enter a financial disclosure statement that describes the sources of funding for the work included in this submission. Review the [submission guidelines](http://journals.plos.org/plosone/s/submission-guidelines#loc-financial-disclosure-statement) for detailed requirements. View published research articles from [PLOS ONE](http://journals.plos.org/plosone/) for specific examples.  This statement is required for submission and will appear in the published article if the submission is accepted. Please make | This research was partially supported by The Koret Fund of Digital Living 2030. This research was partially supported by Ariel Cyber Innovation Center.  The funders had no role in study design, data collection and analysis, decision to publish, or preparation of the manuscript. |

| sure it is accurate. |  |
| --- | --- |
| Unfunded studies Enter: The author(s) received no specific funding for this work. Funded studies Enter a statement with the following details: - Initials of the authors who received each award - Grant numbers awarded to each author - The full name of each funder - URL of each funder website - Did the sponsors or funders play any role in the study design, data collection and analysis, decision to publish, or preparation of the manuscript? - NO - Include this sentence at the end of your statement: The funders had no role in study design, data collection and analysis, decision to publish, or preparation of the manuscript. - YES - Specify the role(s) played. |  |
| * typeset |  |
| Competing Interests  Use the instructions below to enter a competing interest statement for this submission. On behalf of all authors, disclose any [competing interests](http://journals.plos.org/plosone/s/competing-interests) that could be perceived to bias this work—acknowledging all financial support and any other relevant financial or nonfinancial competing interests.  This statement is required for submission and will appear in the published article if the submission is accepted. Please make sure it is accurate and that any funding sources listed in your Funding Information later in the submission form are also declared in your Financial Disclosure statement.  View published research articles from [PLOS ONE](http://journals.plos.org/plosone/) for specific examples. | The authors have declared that no competing interests exist |

| NO authors have competing interests Enter: The authors have declared that no competing interests exist. Authors with competing interests Enter competing interest details beginning with this statement: I have read the journal's policy and the authors of this manuscript have the following competing interests: [insert competing interests here] |  |
| --- | --- |
| * typeset |  |
| Ethics Statement  Enter an ethics statement for this submission. This statement is required if the study involved:   - Human participants - Human specimens or tissue - Vertebrate animals or cephalopods - Vertebrate embryos or tissues - Field research   Write "N/A" if the submission does not require an ethics statement.  General guidance is provided below. Consult the [submission guidelines](http://journals.plos.org/plosone/s/submission-guidelines#loc-guidelines-for-specific-study-types) for detailed instructions. Make sure that all information entered here is included in the Methods section of the manuscript. | The experiment was approved by the Ethical Committee of Tel Aviv university IRB no.  0002152-2, for research proposal given to Dr. Hila Ben-Gal and Prof Irad Ben-Gal. The consent was obtained by a written form and an active action of the participant to approve the consent. |

Format for specific study types

Human Subject Research (involving human

participants and/or tissue)

Give the name of the institutional review

board or ethics committee that approved the

study

•

Include the approval number and/or a

statement indicating approval of this

research

•

Indicate the form of consent obtained

(

written/oral) or the reason that consent was

not obtained (e.g. the data were analyzed

anonymously)

•

Animal Research (involving vertebrate

animals, embryos or tissues)

Provide the name of the Institutional Animal

Care and Use Committee (IACUC) or other

relevant ethics board that reviewed the

study protocol, and indicate whether they

approved this research or granted a formal

waiver of ethical approval

•

Include an approval number if one was

obtained

•

If the study involved

non-human primates

,

add

additional details

about animal welfare

and steps taken to ameliorate suffering

•

If anesthesia, euthanasia, or any kind of

animal sacrifice is part of the study, include

briefly which substances and/or methods

were applied

•

Field Research

Include the following details if this study

involves the collection of plant, animal, or

other materials from a natural setting:

Field permit number

•

Name of the institution or relevant body that

granted permission

•

Data Availability

Authors are required to make all data

underlying the findings described fully

available, without restriction, and from the

time of publication. PLOS allows rare

exceptions to address legal and ethical

concerns. See the

[PLOS Data Polic](http://journals.plos.org/plosone/s/data-availability)

[y](http://journals.plos.org/plosone/s/data-availability)

and

[FA](http://journals.plos.org/plosone/s/data-availability#loc-faqs-for-data-policy)

[Q](http://journals.plos.org/plosone/s/data-availability#loc-faqs-for-data-policy)

for detailed information.

Yes - all data are fully available without restriction

| A Data Availability Statement describing where the data can be found is required at submission. Your answers to this question constitute the Data Availability Statement and will be published in the article, if accepted. |  |
| --- | --- |
| Important: Stating ‘data available on request from the author’ is not sufficient. If your data are only available upon request, select ‘No’ for the first question and explain your exceptional situation in the text box. |  |
| Do the authors confirm that all data underlying the findings described in their manuscript are fully available without restriction? |  |
| Describe where the data may be found in full sentences. If you are copying our sample text, replace any instances of XXX with the appropriate details.   - If the data are held or will be held in a public repository, include URLs, accession numbers or DOIs. If this information will only be available after acceptance, indicate this by ticking the box below. For example: All XXX files   are available from the XXX database (accession number(s) XXX, XXX.).   - If the data are all contained within the manuscript and/or Supporting Information files, enter the following: All relevant data are within the manuscript and its Supporting Information files. - If neither of these applies but you are able to provide details of access elsewhere, with or without limitations, please do so. For example:   Data cannot be shared publicly because of [XXX]. Data are available from the XXX Institutional Data Access / Ethics Committee (contact via XXX) for researchers who meet the criteria for access to confidential data.  The data underlying the results presented in the study are available from (include the name of the third party | The data and code are available in the Github repository of the article. Github address is also found in the reference section.  https://github.com/alonsela2015/Smartphones_QualityOfLife |

| and contact information or URL).  • This text is appropriate if the data are owned by a third party and authors do not have permission to share the data.  * typeset |  |
| --- | --- |
| Additional data availability information: | Tick here if the URLs/accession numbers/DOIs will be available only after acceptance of the manuscript for publication so that we can ensure their inclusion before publication. |

Manuscript	[Click here to access/download;Manuscript;Smartphone](https://www.editorialmanager.com/pone/download.aspx?id=29447015&guid=cbfdaa45-7f1b-4800-863d-4a1081c30319&scheme=1)

[Paper_13.7.21_EthicStateInclude.pdf](https://www.editorialmanager.com/pone/download.aspx?id=29447015&guid=cbfdaa45-7f1b-4800-863d-4a1081c30319&scheme=1)

**Smartphone Use Behavior and Quality of Life: What Is the Role of Awareness?**	1

Alon Sela^1^, Noam Rozenboim^2^, Hila Chalutz Ben-Gal^3^	2

3

1. Department of Industrial Engineering and Management, Ariel University, Ariel,	4

Israel, alonse@ariel.ac.il	5

2. Department of Industrial Engineering, Tel Aviv University, Tel Aviv, Israel.	6
3. Department of Industrial Engineering and Management, Afeka Academic College	7

of Engineering, Tel Aviv, Israel.	8

9

*Abstract* - How does smartphone use behavior affect quality of life factors? The	10

following work suggests new insights into smartphone use behavior,	11

mainly regarding two contradicting smartphone modes of use that affect	12

quality of life in opposite ways. The *Aware* smartphone mode of use	13

reflects an active lifestyle, while the *Unaware* mode of use reflects the use	14

of the smartphone in conjunction with other activities. Using data from 215	15

individuals who reported their quality of life and smartphone use habits,	16

we show that high levels of smartphone use in the Unaware mode of use	17

have a significant negative effect on the quality of life. However, the results	18

show a mild positive effect when the individual uses the smartphone in an	19

aware mode of use. We identify three latent factors within the quality of	20

life construct and measure the effect of the different smartphone modes of	21

use on these quality of life factors. We find that (i) The *functioning* latent	22

factor, which is an individual’s ability to function well in his or her daily	23

life, is not affected by smartphone use behavior. In contrast, (ii) the	24

*competence* latent factor, which is a lack of negative emotions or pain, and	25

(iii) the *positive feelings* latent factor both show a clear effect with the	26

smartphone Unaware mode of use. This implies that the unaware use of	27

smartphones, which is its use in conjunction with other activities or late at	28

night, can be related to lower levels of quality of life. Since smartphones	29

currently serve as an interface between the self and the cyber space, as well	30

as an interface between the self and other individuals online, these results	31

need to be considered for social wellbeing in relation to digital human	32

behavior, smartphone addiction and a healthy mode of use.	33

34

**Keywords:** Smartphone addiction, quality of life, data	35

science, split attention, cognitive load, digital living.	36

37

**1 Introduction**	38

Smartphones are everywhere. As of April 2020, approximately 3.8 billion people in the top	39

fifty most-populated countries used smartphones (Holst, 2020). While some research suggests	40

that smartphone use can be categorized as dangerous (e.g., using a device while driving),	41

inappropriate (e.g., using a device in a cinema or class), or simply excessive (Buchholz, 2016);	42

(Gökçearslan, 2016), smartphones are currently rooted in modern society to such degree that	43

prohibiting or even reducing their use is perceived as a punishment by both adults and younger	44

adolescents.	45

How do various patterns of smartphone use relate to quality of life (QOL) factors? This	46

multidimensional question contains several research branches. The first emphasizes physical	47

and mental health well-being as factors that are influenced by smartphone use (Boase et al.	48

2006; Quan-Haase and Collins 2008; Johnson 2011; Mieczakowski, Goldhaber, and Clarkson	49

2011; Darcin et al. 2016; Lee et al. 2016; McDaniel and Coyne, 2016; Longstreet and Brooks,	50

2017; Rai et al. 2017; Rotondi et al, 2017; Di and Li, 2018). An alternative view focuses on the	51

challenges that smartphones pose for work and family balance (Lilly, Duffy, & Virick, 2006),	52

(Virick, DaSilva, & Arrington, 2010) , as well as stress levels among individuals (Chesley 2005;	53

Wajcman, Bittman, and Brown 2008; Thomée, Härenstam, and Hagberg 2011; Yun et al. 2012;	54

Lee et al. 2014; Wang et al. 2015; Aljomaa et al. 2016; Burrows et al. 2016; Derks et al. 2016;	55

Lee et al. 2016; Park et al. 2016; Škařupová et al. 2016; Zilka, 2018).	56

It seems that the definitions of QOL itself are broad and that there are many methodologies that	57

aim to study this complex but crucial aspect of our lives. QOL research spans the fields of	58

psychology (Land, 1996), philosophy (Brock, 1993), and social psychology (Noll, 2004).	59

Although the QOL literature uses different terms to define the term QOL, it is broadly accepted	60

that QOL can be associated with having a good life (Diener and Suh 1997), e.g., one’s degree	61

of success in reaching a level of satisfaction within the constraints of the resources that one	62

possesses, or factors such as feelings of joy, pleasure, and life satisfaction, which are important	63

constructs of QOL (Land, 1996; Uysal et al. 2016). Additionally, in recent years, the academic	64

discussion about QOL has also included concepts such as work-life balance (Sirgy and Lee,	65

| 2015), which complement the previous QOL measures. The literature that examines the | 66 |
| --- | --- |
| complex interaction between smartphone use and QOL is expansive, but the empirical evidence | 67 |
| regarding the relationship between smartphone use patterns and its consequences for quality of | 68 |
| life are mixed. | 69 |
| In this work, we follow recent calls (Cotten et al. 2019; Mart et al. 2019; Ellis, 2019) and study | 70 |
| QOL factors as a complex phenomenon related to smartphone use behavior. Our goal is to | 71 |
| deepen our understanding of the different effects of smartphone use on QOL to better | 72 |
| understand the contradictory findings. To achieve this goal, we assume that it is not the | 73 |
| smartphone itself but rather the individual’s mental state while interacting with the smartphone | 74 |
| that affects one’s QOL and thus, that it is this type of interaction that should be examined to | 75 |
| study the relationship between smartphone use and QOL. Following this line of study, we | 76 |
| identify two modes of smartphone user behavior, which we define as the *Aware* and *Unaware* | 77 |
| smartphone use modes. | 78 |
| These mental states are defined by in order to settle some contradictory findings in terms of | 79 |
| QOL and states of awareness. For example, Mart et al. (2019) showed that deep levels of | 80 |
| awareness can be associated with higher levels of QOL, while Koch (2004) claimed, | 81 |
| conversely, that low levels of awareness lead to varying levels of QOL. Additionally, it is well | 82 |
| known that when individuals are in low-awareness modes, their ability to accomplish tasks may | 83 |
| suffer (Dinev and Hu, 2010); it is also known that high-awareness modes improve one’s | 84 |
| performance, for example, in medical situations (Bayoumi et al, 2018). These contradictory | 85 |
| effects might impact QOL. We thus measure the relationship between the effect of smartphone | 86 |
| use on QOL in relation with an aware and unaware modes of smartphone operation. | 87 |
| To measure QOL, we use the QOL questionnaire developed by Kreitler and Kreitler (2006). | 88 |
| This is a multidimensional tool that has already been shown to be a valid evaluation | 89 |
| questionnaire for measuring QOL. In this tool, in order to construct the final level of QOL, the | 90 |
| questionnaire measures three different subcomponents that together form the multidimensional | 91 |
| QOL estimates. These subcomponents are named *Competence*, *Functioning* and *Positive* | 92 |

| *Feeling* (see Table 2 for the question construction of these subcomponents). Generally, | 93 |
| --- | --- |
| competence is defined as the presence (or when normalizing, the lack of) negative feelings such | 94 |
| as pain, lack of control, fear, or anxiety. Functioning is defined as feelings of strength, | 95 |
| independence and motivation. Positive feeling is defined as the presence of hope, pleasure, | 96 |
| satisfaction and, to a large degree, the ability to maintain good relationships with others. We | 97 |
| test the relations between these five latent variables (i.e., the three QOL factors and the two | 98 |
| smartphone use modes) using structural equation modeling (SEM) techniques. In addition, as | 99 |
| would be explained, we use conventional statistical methods such as analysis of variance and | 100 |
| dimensionality reduction methods that are borrowed from the data science discipline to | 101 |
| optimize the separation into these subcomponents. | 102 |
| Our empirical results support our hypotheses that high levels of *unaware* smartphone use are | 103 |
| harmful to QOL, while high levels of *aware* smartphone use are not found to significantly affect | 104 |
| QOL parameters. Thus, our findings suggest that it is not simply the magnitude of smartphone | 105 |
| use that needs to be controlled and limited within the effort to balance the negative effect of | 106 |
| excessive and harmful smartphone effect to QOL but rather the mode of operation when one is | 107 |
| using a smartphone and, in particular, the degree to which coactivations with other tasks are | 108 |
| performed during this use. As will be further shown, these coactivations seem to be of high | 109 |
| importance regarding the harmful aspects of excessive smartphone use that negatively affect | 110 |
| QOL. | 111 |
|  | 112 |
| **2 Methods** | 113 |
| **2.1 Data** | 114 |
| We collected 215 questionnaires through Amazon’s Mechanical Turk crowdsourcing | 115 |
| marketplace, which included questions related to both smartphone use habits and quality of life | 116 |
| factors. The Respondents completed the survey task in an average of 5.7 minutes and were paid | 117 |
| 0.5 USD per task. All data, including the questionnaires and the statistical analysis in R and | 118 |

| Python, can be found and downloaded from the GitHub repository related to this work (Sela, | 119 |
| --- | --- |
| n.d.). The gender distribution favored male respondents, with 38% female and 62% male | 120 |
| respondents. Participants’ level of education averaged 15.6 years (SD = 3.15), and the | 121 |
| participants reported having owned their smartphone for an average of 5.48 years (SD = 4.03) | 122 |
| at the time of data collection. Additionally, the participants reported that they had installed an | 123 |
| average of 30 applications on their smartphones. Interestingly, only 13.6% of the female | 124 |
| respondents reported using the device “10 times each hour,” compared to 64% of the male | 125 |
| respondents. In addition, 13% of the female respondents reported using their smartphone | 126 |
| “almost always” while performing another activity, such as driving, cooking or watching | 127 |
| television, compared to 60.7% of the male respondents. Additionally, 22.7% of the female | 128 |
| respondents reported using their phone more than 4 hours on average per day, compared to | 129 |
| 71.4% of the male respondents. | 130 |
| The questionnaire included a shorter version of the QOL factor questions, which were validated | 131 |
| against the full QOL questionnaire (Kreitler and Kreitler, 2006). The questionnaire also | 132 |
| included a set of 12 questions that inspected major smartphone usability habits from three | 133 |
| different aspects of use. The usability questions inspected two types of smartphone use | 134 |
| behaviors. The first type can be broadly generalized as the intensity of use, i.e., the number and | 135 |
| type of applications that a user utilizes while using the phone. The second type consisted of the | 136 |
| user’s mental mode of smartphone use, and the related questions inspected the use of | 137 |
| smartphones in parallel with other user activities. These questions were based on what we | 138 |
| believe could be an important moderating factor in the usability-QOL interaction. The third | 139 |
| type of question included the user’s report on his/her beliefs of his/her addictive use behavior, | 140 |
| as well as the degree to which he/she believes that a smartphone is an indispensable part of | 141 |
| one’s social and professional life. | 142 |
| From the initial 215 surveys that were collected, 4 surveys were removed due to the poor quality | 143 |
| of data within. In the remaining 211 surveys, with respect to the respondents’ occupations, 74% | 144 |
| reported working full-time jobs, and the rest reported working either part-time or not working | 145 |
| at all. A total of 98% reported having one single phone, and the rest reported having two or | 146 |

| even three phones. Last, all the participants reported that they had owned their phone for at | 147 |
| --- | --- |
| least six months. This question was important to ensure that the participants had owned a phone | 148 |
| long enough for its use to have impacted their lives. | 149 |
| **2.2 Quality of Life Measurement** | 150 |
| We based the QOL measurement on the Multidimensional Quality of Life Inventory (MQOL) | 151 |
| questionnaire (Kreitler and Kreitler, 2006). This scale includes twenty-two items that represent | 152 |
| fifteen different domains of QOL: functioning in the family, physical health, living conditions, | 153 |
| sexuality, body image, cognitive functioning, work and profession, social functioning, presence | 154 |
| of positive emotions, presence of negative emotions, meaningfulness of life, confusion and | 155 |
| bewilderment, ability to cope and expression of stress. A higher score represents better QOL | 156 |
| results. Additionally, sections of the questionnaire include measures related to psychographic | 157 |
| details, stress levels and demographic and control variables. | 158 |
| **2.3 Hypotheses and Latent Variables Construction** | 159 |
| We started by analyzing the data to determine how to best separate the variables into their latent | 160 |
| subcomponents, i.e., latent variables for both QOL and smartphone usability. These latent | 161 |
| variables were then employed in our model and in the questionnaires. According to our | 162 |
| preliminary analyses and based on dimensionality reduction methods borrowed from the data | 163 |
| science discipline (Suhr, 2006), (Zwick & Velicer, 1986), (Peterson, 2000), we separated the | 164 |
| QOL questions into three main subcomponents (latent variables) and the smartphone use | 165 |
| questions into two latent variables (see Table 1 and Table 2 below for more details). In | 166 |
| accordance with the QOL subcomponents developed by Kreitler and Kreitler (2006), we named | 167 |
| the three latent QOL variables *'competence,' 'functioning,' and 'positive feeling*.' Furthermore, | 168 |
| we named the two smartphone usability latent variables *'aware’* and *'unaware’* use behavior | 169 |
| modes. The method used to separate the latent variables is further described in the results | 170 |
| section and in Figure 2, Figure 3, Table 1 and Table 2. Further descriptions of each latent | 171 |
| variable are provided below. | 172 |

| ***Unaware smartphone use*** - Unaware smartphone use represents smartphone use that is | 173 |
| --- | --- |
| simultaneously performed with other activities that demand attention, such as cooking, driving | 174 |
| and watching television. Similarly, an important component of unaware use is the use of a | 175 |
| smartphone in the middle of the night, where one’s level of attention is usually lower due to | 176 |
| tiredness; thus, the activities performed while using the smartphone may be associated with its | 177 |
| use while the individual is expressing lower levels of concentration. | 178 |
| ***Aware smartphone use -*** This smartphone use is characterized by actively utilizing the numerus | 179 |
| functionalities of the smartphone device, including its built-in features, for example, using its | 180 |
| camera and calculator or the intensive use of email, calendar, notetaking, etc. These smartphone | 181 |
| functionalities might reflect an active and busy lifestyle. Note that the two use modes do not by | 182 |
| nature contradict each other; theoretically, a person might show high levels of aware use | 183 |
| together with high levels of unaware use at the same time. | 184 |
| ***Competence*** - The competence latent variable refers to an individual's perception of the degree | 185 |
| to which they believe they can cope with life. This latent variable is also associated with QOL | 186 |
| items that reflect the existence of negative feelings, such as pain, a sense of lack of control of | 187 |
| one’s life, a sense of uncertainty, and feelings of anxiety, fear, depression, sadness, tension, and | 188 |
| restlessness. | 189 |
| Following the definitions above, we hypothesize the relationship between *smartphone use* and | 190 |
| the *competence* latent variable of QOL as follows: | 191 |
| **H1.** *Higher levels of unaware smartphone use are associated with lower levels of* | 192 |
| *competence.* | 193 |
| **H2.** *Higher levels of aware smartphone use are associated with higher levels of competence.* | 194 |
| ***Functioning*** - The functioning latent variable in QOL (Kreitler and Kreitler, 2006) examines | 195 |
| whether a person functions well in his/her basic daily routine, both at home and at work. This | 196 |
| latent variable refers to numerus attributes of regular daily and life functioning dimensions, | 197 |
| such as one’s healthy physical functioning, a good level of functioning within the family, | 198 |

| normal cognitive abilities, e.g., the ability to concentrate and the ability to solve problems, and | 199 |
| --- | --- |
| one’s ability to keep healthy relationships with family and friends. It also includes QOL items | 200 |
| such as the ability to sustain a job, the performance of regular physical exercise, the ability to | 201 |
| perform basic life activities such as eating, sleeping, or taking care of yourself, and in general, | 202 |
| the feeling of being able to cope with the basic requirements of one’s daily routine of life. | 203 |
| We believe that being capable of using numerus and technically complex applications on a | 204 |
| smartphone, such as texting, using reminders and notes, using GPS-based location services or | 205 |
| using social media, should be positively correlated with the functioning latent variable of QOL. | 206 |
| Technical competence in using complex applications should reflect (to some degree) basic | 207 |
| cognitive functioning, and these basic cognitive functioning are expected to improve one’s | 208 |
| ability to perform basic daily tasks. While the use of numerus complex applications should | 209 |
| reflect the normal functioning of basic cognitive skills and support a better QOL, we also | 210 |
| suspect the existence of a contradicting effect in which an overuse of numerus complex | 211 |
| applications on one’s smartphone might be a sign of cognitive overload. Thus, due to this effect, | 212 |
| such overuse is expected to be correlated with lower levels of the functioning latent variable | 213 |
| and lower levels of QOL. Therefore, we hypothesize as follows: | 214 |
| **H3.** *Higher levels of unaware smartphone use are associated with lower levels of the* | 215 |
| *functioning latent variable of QOL.* | 216 |
| **H4.** *Higher levels of aware smartphone use are associated with higher levels of the* | 217 |
| *functioning latent variable of QOL.* | 218 |
| ***Positive Feeling*** – The positive feeling latent variable in QOL measurement (Kreitler and | 219 |
| Kreitler, 2006) examines the degree to which an individual experiences positive emotions. It is | 220 |
| to some degree the opposite of the competence latent variable, in which one feels helpless, | 221 |
| depressed and incompetent about one’s life. It also differs from the functioning variable, which | 222 |
| is more external and measures one’s functionality and instead of one’s emotions. That is, an | 223 |
| individual can be fully functioning but also depressed (high levels of functioning and low levels | 224 |

| of competence). Similarly, we expect it to be less likely that one would experience high levels | 225 |
| --- | --- |
| of positive feeling and high levels of competence at the same time. The positive feeling variable | 226 |
| is characterized by QOL items that refer to the regular experience of positive health, a general | 227 |
| sense of hope and the ability to feel pleasure. It also includes questions related to an ability to | 228 |
| maintain healthy relationships with one’s associates, friends and intimate partners. | 229 |
| We hypothesize that intensive use of a smartphone for communication purposes helps with | 230 |
| one’s everyday communication with friends and family and thus improves one’s overall QOL | 231 |
| satisfaction and level of positive feelings. We also believe that intensive unaware smartphone | 232 |
| use, among other things, might determine the degree to which one is “using a smartphone in | 233 |
| the middle of the night,”, thus, might lead to sleep deprivation and reflect signs of addictive | 234 |
| smartphone behavior. These could lead to lower levels of the positive feeling latent variable | 235 |
| and to overall lower levels of QOL. Thus, we define two additional hypotheses related to the | 236 |
| relationship between smartphone use and the QOL positive feeling latent variable. These are | 237 |
| defined as follows: | 238 |
| **H5.** *Higher levels of unaware smartphone use are associated with lower levels of the positive* | 239 |
| *feeling latent variable of QOL.* | 240 |
| **H6.** *Higher levels of aware smartphone use are associated with higher levels of the positive* | 241 |
| *feeling latent variable of QOL.* | 242 |
| The six hypotheses above are summarized in the illustration below. These hypotheses will be | 243 |
| examined and evaluated by utilizing the structural equation modeling method (SEM) | 244 |
| (Rosseel, 2012), which is implemented through the Lavaan package in R (Rosseel, 2012). | 245 |

| **Figure** 1. Proposed Theoretical Model and Hypotheses | 246 |
| --- | --- |
| **2.4 Ethics statement** | 247 |
| The experiment was approved by the Ethical Committee of Tel Aviv university IRB | 248 |
| for research proposal no. 0002152-2, given to Dr. Hila Ben-Gal. The participant | 249 |
| consent informed was written and they were informed by the statement “*We are* | 250 |
| *conducting an academic survey about smartphone usage habits and other aspects of* | 251 |
| *life. We need to understand your opinion about the questions in the survey. Press the* | 252 |
| *link below if you agree to participate in the survey*”. Only users that actively pressed | 253 |
| the link after reading this statement could participate in the survey. After completing | 254 |
| the survey, each responder was paid 0.5 USD from the Amazon Mechanical Turk | 255 |
| tasks system. | 256 |
| **3 Results** | 257 |
| **3.1 Defining Latent Variables by Dimensionality Reduction** | 258 |
| In order to define the best constructs of the different sub components, one common method that | 259 |
| is used in data science to determine latent variables and construct higher-level hierarchical | 260 |
| classes from individual questions is the use of dimensionality reduction. According to this | 261 |

| approach, the elbow method (Suhr, 2006) is commonly used as a practiced heuristic that helps | 262 |
| --- | --- |
| to determine, based on the data alone, the best number of latent variables that represent the | 263 |
| exploratory factor analysis (Thompson, 2004) (Flora, 2017). | 264 |
| Following to this method, we first used the elbow method on an x-y plot, where the number of | 265 |
| latent variables (x-axis) was plotted against the corresponding eigenvalues (y-axis). These | 266 |
| eigenvalues represent the additional variance that can be explained from the total variance of | 267 |
| different methods of separation into lower dimensions. The plot usually has a large negative | 268 |
| derivative at the first eigenvalues, which then quickly decays as more dimensions (eigenvalues) | 269 |
| are introduced into the model. This process creates a plot with an elbow shape, (see Figure 2) | 270 |
| with the breakpoint of the slope, i.e., the elbow, representing the optimal number of latent | 271 |
| variables that best fit the model. We used this method to choose the best number of latent | 272 |
| variables for the smartphone use dimension, as well as for the QOL variable. We considered | 273 |
| using other methods, such as the Kaiser criterion (Kaiser, 1960) and Horn's parallel analysis | 274 |
| (Horn, 1965); however, we chose to use the elbow method mainly because of its good results, | 275 |
| common usage and simplicity (Zwick & Velicer, 1986). | 276 |
| The best number of latent variables that separates each of the two major factors, i.e., smartphone | 277 |
| use and QOL, is presented in Figure 2. The left image presents the optimal number of latent | 278 |
| variables for the phone usability factor. According to this method, the optimal number of | 279 |
| subcomponents for smartphone usability is 2 components (left image). For the QOL factor, the | 280 |
| optimal number of components is 3 latent variables (right image). | 281 |
|  | 282 |

| **Figure 2.** The number of latent variables was determined by the elbow method. These plots show the number of | 283 |
| --- | --- |
| latent variables associated with the smartphone use modes (left) and quality of life (right). The x-axis is the number | 284 |
| of latent variables, and the y-axis is the additional variance that can be explained for each additional latent variable. | 285 |
| Note that for the phone use factor, the elbow method determined 2 latent factors as an effective separation, while for | 286 |
| the QOL factor, it determined 3 latent factors. | 287 |
|  | 288 |
| **3.2 Inner Correlations of Smartphone Use and QOL** | 289 |
| To understand and validate the interaction between smartphone use behavior and QOL factors, | 290 |
| we also analyzed a correlation matrix of the entire data set. Figure 3 below presents three | 291 |
| distinct zones in the full correlation matrix. The lighter (yellow) color represents a lower | 292 |
| correlation, while the green color represents a higher correlation. Zone A shows the inner | 293 |
| correlation within the smartphone use behavior questions. As illustrated by the green square in | 294 |
| A, users who scored high on some dimensions of smartphone use also tended to score high on | 295 |
| the others. Similarly, zone B presents the inner correlation matrix within the QOL factors. | 296 |
| Similarly, inner validity is well observed in zone B. Finally, zone C represents the correlation | 297 |
| matrix between smartphone use behavior and QOL factors. However, as illustrated in the | 298 |
| image, these correlations are not as strong as the inner correlations within each topic (i.e., zone | 299 |
| A and zone B), which, due to their relatively stronger inner correlation, change the entire image | 300 |
| color to a greenish color; the QOL usability matrix in zone C seems to be weaker in comparison. | 301 |
| **3.3 Determining the Questions that Best Construct Each Latent Variable** | 302 |
| In the previous section, we determined the best number of latent variables for QOL and | 303 |
| smartphone usability. Next, we needed to define what questions should be assigned to each | 304 |
| latent variable. Deciding on the latent variables required a preliminary analysis that determined | 305 |
| which observed variables (questions) belong to each latent variable. We performed this analysis | 306 |
| using the observed variable loading method (Peterson, 2000). According to this method, the | 307 |
| loading factors of each question represent the relationship of each question to the underlying | 308 |
| latent variable (Lior & Maimon, 2005), (Maimon & Rokach, 2005). The loading value is the | 309 |
| correlation coefficient between the observed variable and the latent variable. The higher the | 310 |
| loading factor is for each question and latent variable, the better the question represents the | 311 |
| latent variable. According to this method, loading value of under 0.3 means very weak | 312 |
| correlations that should be ignored. This analysis and the item loading results are presented in | 313 |
| Table 1 for smartphone use and in Table 2 for QOL. Note that the green-colored cell signifies | 314 |
| the binary decision regarding the best allocation of each question to the most suitable latent | 315 |
| variable. | 316 |

 317

**Figure 3.** Correlation matrix of questionnaire items. Green indicates a strong correlation, and red/yellow indicates a 318 weaker correlation. Smartphone use behavior (A) and quality of life items (B) show a good separation, which forms 319 two main clusters of items, one related to QOL and the other to smartphone use behavior. (C) shows weaker 320

relationship in comparison to each individual sub-component.	321

322

**Table 1.** Smartphone Use Items	323

| **Variable** | **Smartphone Use Items** | **Aware** | **Unaware** |
| --- | --- | --- | --- |
| x1 | How many applications that are being used do you have on your smartphone/cellular phone? | -0.18 | 0.64 |
| x2 | Do you use your smartphone/cellular phone for activities that can be performed with other means, for example, camera, calculator, notes, reading books, making payments? | 0.1 | 0.4 |
| x3 | Do you go to bed with the smartphone/cellular phone at your side? | 0.44 | 0.12 |
| x4 | Do you use your smartphone/cellular phone in the middle of the night? | 0.85 | -0.2 |
| x5 | Do you use your smartphone/cellular phone in parallel with other activities, such as driving, cooking, watching TV? | 0.73 | -0.02 |
| x6 | Do you take your smartphone/cellular phone with you to the restroom? | 0.45 | 0.05 |
| x7 | Frequency of using your smartphone/cellular phone: on average, how many times do you use it each hour during the day? | 0.11 | 0.4 |
| x8 | Do you use the location-based services of the smartphone/cellular phone? | 0.12 | 0.4 |
| x9 | How many social networks (e.g., Facebook, Twitter) are downloaded on your smartphone/cellular phone? | 0.03 | 0.56 |
| x10 | Do you consider your smartphone/cellular phone to be an indispensable tool for your work? | 0.52 | -0.06 |
| x11 | Do you consider your smartphone/cellular phone to be an indispensable tool for your social life? | 0.52 | 0.08 |
| x12 | Do you consider yourself to be addicted to the use of your smartphone/cellular phone? | 0.47 | 0.17 |

324

**Table 2.** Quality-of-life Items	325

| **Variable** | **Quality-of-life Item** | **ning** | **Functio**  **ence** | **Compet**  **Feeling** | **Positive** |
| --- | --- | --- | --- | --- | --- |
| Y1 | A general sense of good physical health |  | 0.17 | 0.01 | 0.38 |
| Y2 | Lack of pain |  | -0.04 | 0.61 | 0.02 |

|  | Y3 | Work (outside the home or in the home, including household work) | 0.43 | -0.15 | 0.14 |  |  |
| --- | --- | --- | --- | --- | --- | --- | --- |
|  | Y4 | Activity (outside the home or inside the home, not including work activity) | 0.34 | -0.1 | 0.25 |  |  |
|  | Y5 | Strength and ability to perform the activities of eating, sleeping, etc. | 0.8 | 0.04 | -0.02 |  |  |
|  | Y6 | Strength and ability to perform activities within the family (as a partner, parent, sibling, son/daughter) | 0.41 | 0.06 | 0.32 |  |  |
|  | Y7 | Intimate relations with your partner | -0.01 | -0.05 | 0.61 |  |  |
|  | Y8 | Relations with your friends, acquaintances, relatives | 0.09 | 0.04 | 0.45 |  |  |
|  | Y9 | [Taking care of yourself and your external appearance | 0.49 | -0.06 | 0.27 |  |  |
|  | Y10 | Strength and ability to cope with the tasks of your everyday life | 0.81 | 0.12 | -0.03 |  |  |
|  | Y11 | Independence in functioning and activity in your daily life | 0.78 | 0.07 | -0.04 |  |  |
|  | Y12 | Sense of control over situations, feeling that you can determine what happens | 0.38 | 0.53 | -0.34 |  |  |
|  | Y13 | Ability to concentrate on the task you are performing | 0.76 | -0.04 | 0 |  |  |
|  | Y14 | Ability to think, solve problems | 0.76 | -0.04 | 0 |  |  |
|  | Y15 | Sense of certainty, clarity | 0.1 | 0.65 | -0.04 |  |  |
|  | Y16 | Anxiety, fear (lack of) | -0.06 | 0.74 | 0.15 |  |  |
|  | Y17 | Depression, sadness (lack of) | -0.12 | 0.84 | 0.17 |  |  |
|  | Y18 | Tension, restlessness (lack of) | -0.22 | 0.8 | 0.24 |  |  |
|  | Y19 | Hope | 0.09 | 0.02 | 0.63 |  |  |
|  | Y20 | Pleasure | -0.07 | 0.13 | 0.81 |  |  |
|  | Y21 | Motivation to make efforts and continue doing things | 0.48 | 0.04 | 0.33 |  |  |
|  | Y22 | Satisfaction with life in general | 0.2 | 0.18 | 0.56 |  |  |
|  | | | | | | | 326 |
| **3.4 Model Evaluation** | | | | | | | 327 |
| To evaluate the fit of our model to SEM, we tested our model fit using the following criteria | | | | | | | 328 |
| (Hu, 1995): comparative fit index; CFI = .934; Tucker-Lewis index; TLI = 0.928; root mean | | | | | | | 329 |
| square error of approximation; RMSEA = 0.004; and standardized RMR SRMR = .06. These | | | | | | | 330 |
| criteria suggested that SEM could be safely applied to our data. | | | | | | | 331 |
| Additionally, we performed an explanatory factor analysis to validate the fitness of the different | | | | | | | 332 |
| latent variables. As seen in the CFA results (see section I in the Supplementary Materials | | | | | | | 333 |
| section), we achieved excellent fit indices. While at first glance this excellent fit might seem | | | | | | | 334 |
| unclear, or even suspicious, one needs to consider that the method used in this analysis | | | | | | | 335 |
| determined the latent factors from the data itself through the dimensionality reduction method | | | | | | | 336 |
| and not by making a predominant assumption about these latent variables. Because we first | | | | | | | 337 |
| found the optimal number of latent variables and then allocated each question to the most | | | | | | | 338 |

| adequate latent variable, our use of this method and the subsequent allocation of the questions | 339 |
| --- | --- |
| to the latent variable resulted in an almost perfect CFA. Indeed, in many cases in the social | 340 |
| sciences, the theory used leads the separation of the questions into different latent variables. | 341 |
| We took the data science path and first analyzed the data. Then, through this initial analysis, | 342 |
| we found new possible insights based on the data and then supported them statistically. | 343 |
|  | 344 |
| **3.5 Structural Equation Modeling Analysis Results** | 345 |
| To evaluate our hypotheses, we used structural equation modeling (SEM) package in the | 346 |
| Lavaan Package of R Software) (Rosseel, 2012). Figure 4 shows the connections among the | 347 |
| different latent variables in our model, with red arrows representing negative coefficients and | 348 |
| green arrows representing positive coefficients. The values on the arched links are the | 349 |
| normalized correlations between different latent variables of the same factor, while the values | 350 |
| on the straight lines are the regression coefficients between the source and the target. In the first | 351 |
| model, we analyzed the relations between the smartphone use modes (aware and unaware) and | 352 |
| the three latent variables that form QOL (competence, positive feeling and functioning). We | 353 |
| measured both the direct model, in which we inspected the effects of smartphone usability on | 354 |
| QOL in Figure 4 (left), and the inverse model, in which we inspected the inverse effects of | 355 |
| OQL on usability in Figure 4 (right). Overall, the entire SEM model had good results, with a | 356 |
| comparative fit index (CFI) = 0.934; a Tucker-Lewis index (TLI) = 0.928; a root means square | 357 |
| error of approximation (RMSEA) = 0.004; and a standardized RMR (SRMR) = 0.06. | 358 |

| **Figure 4. SEM Models: Smartphone usability effects on QOL (left) and its inverse model, effects of QOL on** | 359 |
| --- | --- |
| **usability (right).** For the direct model (left), unaware use (unw) negatively effects all three QOL measures: | 360 |
| competence (cmp), functioning (fnc) and positive feeling (ps_), while aware use (awr) positively effects positive | 361 |
| feelings (ps_) but does not significantly affect the functioning or competence latent variables. For the inverse model | 362 |
| (right), the functioning (fnc) QOL latent variable negatively effects both aware (awr) and unaware (unw) usability, | 363 |
| while competence does not significantly affect either of the latent variables of aware or unaware use; furthermore, | 364 |
| positive feelings (ps_) positively effects the aware component of QOL but not the unaware component. | 365 |
|  | 366 |
| The direct SEM model found support for the relationships between the QOL factors and phone | 367 |
| use. This model implies a strong connection between smartphone use and QOL; however, it | 368 |
| could be that it is the QOL that affects smartphone use and not the. opposite Figure 4 (right) | 369 |
| demonstrates the opposite direction model. High inverse estimates (with p-value > 0.05) can be | 370 |
| found between functioning (fnc) and unaware (unw) with correlation of -0.77, and functioning | 371 |
| (fnc) and aware (awr) with correlation of -0.52. Additionally, strong correlations are found | 372 |
| between positive feelings (ps_) and aware (awr) with correlation of 0.91. The rest of the | 373 |
| estimates are very low and therefore show an insignificant connection between the additional | 374 |
| latent variables. | 375 |
|  | 376 |
| **Table 3.** Summary of the Structural Model Estimates and P-Values. | 377 |

**Quality of Life**

*Regressions	Hypothe Estimates	SE	P(>|z|)	Std.all*	**Hypothesis**

|  | Competence – Unaware | H1 | -0.409 | 0.14 | **0.05** | -0.505 | Support | |  |
| --- | --- | --- | --- | --- | --- | --- | --- | --- | --- |
|  | Competence – Aware | H2 | 0.485 | 0.32 | 0.136 | 0.290 | Reject | |  |
|  | Functioning – Unaware | H3 | -0.322 | 0.11 | **0.004** | -0.538 | Support | |  |
|  | Functioning – Aware | H4 | 0.289 | 0.23 | 0.210 | 0.234 | Reject | |  |
|  | Positive Feeling – Unaware | H5 | -0.496 | 0.15 | **0.001** | -0.694 | Support | |  |
|  | Positive Feeling – Aware | H6 | 0.929 | 0.36 | **0.011** | 0.629 | Support | |  |
| *N=211* | | | | | | | |  | |
|  | | | | | | | | 378 | |
| Our analysis yields a complex set of relationships between smartphone use and QOL. | | | | | | | | 379 | |
| Nevertheless, if we aggregate the two images and only consider the supported hypotheses, as | | | | | | | | 380 | |
| presented in Table 3, we can conclude that using a smartphone in the unaware mode is likely | | | | | | | | 381 | |
| to negatively influence all three components of one’s QOL, while having a strong positive | | | | | | | | 382 | |
| feeling component about one’s QOL is likely to positively affect the aware mode of use of | | | | | | | | 383 | |
| one’s smartphone. | | | | | | | | 384 | |
|  | | | | | | | | 385 | |
| **3.6 Deeper Inspection of the Unaware Smartphone Use Mode** | | | | | | | | 386 | |
| To further understand the relationship between the unaware smartphone mode of use and QOL, | | | | | | | | 387 | |
| we further inspected the effect of the unaware latent variable of QOL on each question. Note | | | | | | | | 388 | |
| that for each of the 7 questions that form the unaware latent variable, the respondents were | | | | | | | | 389 | |
| asked to grade the degree to which they agreed with each statement about themselves on a scale | | | | | | | | 390 | |
| ranging from 0 to 2. The person’s unaware mean score, denoted as 𝑆_𝑈_̅ , can thus be computed | | | | | | | | 391 | |
| as the mean of these 7 answers. We separated the population into 3 separate groups according | | | | | | | | 392 | |
| in their average unaware score. We ignored the middle group and only inspected the users with | | | | | | | | 393 | |
| high and low unaware scores, which were denoted as the 𝐻⃗ and 𝐿⃗ groups, respectively. | | | | | | | | 394 | |
| By separating these groups, we were able to directly look for significant differences in the levels | | | | | | | | 395 | |
| of QOL and compare the QOL of users who had high levels of unaware smartphone use with | | | | | | | | 396 | |
| those who had low levels. Furthermore, we were able to look at each QOL component directly | | | | | | | | 397 | |

| between these groups. Figure 5 shows the distribution of QOL, as well as the 3 separate QOL | 398 |
| --- | --- |
| components, between these two groups. The positive feeling subcomponent had the strongest | 399 |
| level of separation between the 𝐻⃗ and 𝐿⃗ groups (t-test, p-value<0.02), while the functioning | 400 |
| subfactor difference was not significant (t-test, p-value = 0.23). We can therefore conclude that | 401 |
| when measured directly, users with a low level of unaware smartphone use (blue) are likely to | 402 |
| experience higher levels of competence and experience stronger levels of positive feelings, | 403 |
| while the effect on functioning, although in a similar (negative) direction, is less clear. | 404 |

|  | 405 |
| --- | --- |
| **Figure 5 .** Distribution of QOL scores for high unaware (orange) vs. low unaware smartphone use (blue). In (a), we | 406 |
| plot the histograms for the entire QOL component. In (b), we plot only the positive feeling subcomponent. In (c) we | 407 |
| only plot the competence subcomponent. Finally, in (d), we plot only the functioning subcomponent. The exact | 408 |
| questions that construct each QOL subcomponent can be seen in Table 2, while the questions that construct | 409 |
| smartphone usability are found in Table 3. | 410 |
|  | 411 |

| **4 Discussion** | 412 |
| --- | --- |
| The ever-growing importance of technology and smartphones in modern society is clear (Park | 413 |
| and Lee, 2012). Smartphones have been even more important during COVID-19 lockdowns | 414 |
| and quarantines, which require long stay-at-home routine, while connecting remotely to work, | 415 |
| friends and family. In this article, we attempted to deepen our understanding of the effect of | 416 |
| smartphone use behavior on different QOL factors. Following some disagreement regarding | 417 |
| technology use behavior and our belief that it is not the technology itself but rather the | 418 |
| interaction between individuals and the technology that may affect humans, both to positive or | 419 |
| to negative feelings, we focused on the interface between humans and technology. Thus, this | 420 |
| study analyzed specific smartphone use patterns to better understand the modes in which people | 421 |
| use smartphones and how these modes contribute to QOL factors. We identified two main | 422 |
| smartphone use behavior modes, namely, the aware and unaware modes of use. We also defined | 423 |
| three QOL factors, named competence, functioning, and positive feeling. | 424 |
| We try to solve the disagreement between the claims that smartphones effects QOL positively, | 425 |
| and those claiming its negative effect, by suggesting two opposite use effects on QOL. We | 426 |
| found that while intensive use in the aware mode reflects an active lifestyle and contributes to | 427 |
| positive levels of QOL, the unaware mode of use contributes to an opposite effect and results | 428 |
| in reduced levels of QOL. | 429 |
| Looking at the relationships between the different subcomponents of smartphone use and the | 430 |
| different subcomponents of QOL, we found that the strongest relationship is the negative effect | 431 |
| between smartphone unaware use and the positive feeling subcomponent of QOL (p=0.001). | 432 |
| The strong effect of unaware use may be explained through the concept of *cognitive loading* | 433 |
| (Sweller, J., 2011; Paas et al., 2003), which is a “mechanistic” theory that assumes that human | 434 |
| cognitive resources are limited and thus, when these limited resources are distributed between | 435 |
| too many tasks, performance is thereby reduced. This theory is also supported in the context of | 436 |
| FMRI studies (Szameitat, 2002), in which cognitive loading can be directly observed and | 437 |
| measured by analyzing changes in brain images. While FMRI methods can directly observe | 438 |

| cognitive loading, the commonly used method to measure cognitive loading inspects the | 439 |
| --- | --- |
| decrease in the performance of respondents when they are asked to simultaneously perform | 440 |
| dual (or multiple) tasks (Brünken, 2004). One should note that in our study, smartphone users | 441 |
| were required to report whether they used their smartphone in parallel with other activities. We | 442 |
| thus expected higher mental loadings (Botzer, Meyer, & Parmet, 2016) when smartphones were | 443 |
| used in parallel with other activities. Cognitive loading is also known to increase stress levels | 444 |
| (Setz, 2009), which results in aggression and negatively affects the ability of an individual to | 445 |
| maintain good relationships with others. Relationships with others, on the other hand, are a | 446 |
| major part of the positive feeling subcomponent in the QOL questionnaire (see Table 2). We | 447 |
| thus see a direct link between the parallel performance of different tasks and smartphone use, | 448 |
| which affects mental loadings, which contribute to stress and, in the long term, can harm one’s | 449 |
| good relationships with others and thereby reduce the positive feeling subcomponent of QOL. | 450 |
| Unaware smartphone use in conjunction with other activities may also be associated with | 451 |
| addiction. For example, the examination of other types of technological addictions (e.g., | 452 |
| internet addiction) has found that one well-validated predictive variable for internet addiction | 453 |
| is a high level of attention deficit disorder (ADD) (Panagiodity, 2018). It is possible that people | 454 |
| with some level of ADD might experience a higher tendency toward smartphone addictive use | 455 |
| patterns, which in turn should reduce their level of the positive feelings QOL component. As | 456 |
| demonstrated in Figure 4, the level of the positive feelings component does not reduce the | 457 |
| results of the unaware QOL component, but high level of the unaware QOL component does | 458 |
| reduce the positive feelings QOL component. This one-directional influence may suggest | 459 |
| causality. In contrast, the relationship between aware smartphone use and the positive feelings | 460 |
| QOL component is bidirectional, which indicates correlation and not causation. Thus, our | 461 |
| results support the growing level of concern about the possible harmful mental implications of | 462 |
| the excessive use of technological devices (Škařupová et al. 2016) while also focusing on the | 463 |
| unaware mode of use. | 464 |
| The theory of planned behavior (TPB) (Ajzen, 1991) may partially explain our results from | 465 |

| another perspective. According to the TPB, behavior is determined by an individual’s intentions | 466 |
| --- | --- |
| to perform the behavior. Intention is influenced by (i) attitude, (ii) personal subjective norms, | 467 |
| and (iii) perceived behavioral control (PBC), which is one’s ability to mindfully control one’s | 468 |
| behavior. These three components together directly impact behavior. Individuals may diverge | 469 |
| in their smartphone use behavior in at least two analytically distinct ways, i.e., when they use | 470 |
| smartphones in the aware mode and when they use it in the unaware mode. According to our | 471 |
| results, using smartphones sometimes involves aware and purposeful activities (e.g., reading a | 472 |
| book, searching the web) and sometimes involves unaware, unmindful usage. Thus, the TPB | 473 |
| may explain an individual’s active decisions and preferred choices regarding the utilization of | 474 |
| his or her time, which results in the awareness state and thereby negatively influences some | 475 |
| QOL features. | 476 |
| To conclude, while an active lifestyle in which smartphones play an important role is likely to | 477 |
| improve one’s overall level of satisfaction and QOL factors, our research findings indicate a | 478 |
| possible domain in which this effect might be harmful when one’s smartphone use is performed | 479 |
| in parallel with other activities. Therefore, this work increases the need to differentiate between | 480 |
| smartphone use modes for the benefit of users and society. | 481 |
|  | 482 |
| While we believe that this work presents promising avenues for future research and practice, | 483 |
| we are also cognizant of its limitations. First, despite an acceptable response rate (Baruch and | 484 |
| Holtom 2008), we are aware of the limitations of utilizing the Mechanical Turk open-source | 485 |
| data collection tool, although this approach has been seen to have practical and methodological | 486 |
| benefits (Cheung et al. 2017). | 487 |
| Second, our data included a mid-range number of participants, and it is important to keep in | 488 |
| mind that our empirical setting involved individual smartphone users who provided self-report | 489 |
| data on the Mechanical Turk online platform. Questions therefore remain about how our results | 490 |
| can be generalized to other contexts, such as wider aspects of society and the general | 491 |
| population, for which the focus may differ. Additionally, we believe there is a need to | 492 |
| complement the existing survey-based measures, which provide mostly static (or at best | 493 |

| episodic) snapshots of QOL over an individual’s life cycle (see, for example, Elhai et al, 2017; | 494 |
| --- | --- |
| Buhrmester et al, 2011) and thus may be susceptible to various forms of self-report bias. | 495 |
|  | 496 |
| **5 Conclusion** | 497 |
| This study examines an unsolved debate regarding the contradictory effects of smartphone use | 498 |
| on quality of life. While prior studies have reported mixed and contradicting effects regarding | 499 |
| the relationship between smartphone use and QOL, our main contribution to the field is our | 500 |
| inspection of the problem through the lens of the mental states of the users while they are | 501 |
| utilizing the technology. By separating user behavior into two distinct mental states, namely, | 502 |
| aware use and unaware smartphone use, we seem to successfully address the contradiction | 503 |
| noted above. Although the use of smartphones in a highly aware state reflects an active lifestyle | 504 |
| in which one is likely to feel competent and functioning and to experience positive feelings | 505 |
| about one’s life, smartphone use in an unaware state is likely to reflect some lack of | 506 |
| mindfulness, the absence of concentration, an overload, or similar mental states that can be | 507 |
| associated with an addictive and unmindful state of mind. These mental states might also go | 508 |
| along higher levels of negative emotions, which the user tries to compensate through an | 509 |
| excessive levels of smartphone use and sensory feedback, probably unsuccessfully. | 510 |
| In our digital era, we are all addicted to our smartphones to some degree. Whether this addiction | 511 |
| is harmful seems to be mediated by the mental modes in which we use the smartphone. Whether | 512 |
| we use a smartphone while in an active concentrated and focused frame of mind or its use while | 513 |
| in an absent-minded and split-awareness frame of mind, seems to determine its outcomes to the | 514 |
| QOL. | 515 |
| During the COVID-19 pandemic, more than ever before, larger parts of our private and | 516 |
| professional lives have been performed through the internet and/or through smartphones. | 517 |
| Additionally, the clear separation between our personal and private lives has eroded as people | 518 |
| have been forced to work from their homes. Based on our findings, we believe this shift to a | 519 |
| remote work-from-home format could potentially worsen the amount of the unaware use of | 520 |

| smartphones, since people use smartphones in an ever-growing multi-tasking. We believe that | 521 |
| --- | --- |
| nowadays, more than before, smartphones are not only a gateway that connects individuals to | 522 |
| the other individuals in the external world, but are also used in performing numerous tasks | 523 |
| related to both family and work. As a result, these tasks are now blended together in the lives | 524 |
| of working-from-home or quarantined workers. Indeed, the worsening of several mental health | 525 |
| parameters has been reported and has been associated with excessive smartphone use (Garfin, | 526 |
| 2020), (Elhai et al 2020). This excessive behavior related to unaware smartphone use may result | 527 |
| in negative effects, such as stress, fatigue, and addictive online behavior. A better understanding | 528 |
| of the smartphone uses modes and, in particular, their association with QOL in regard to remote | 529 |
| work scenarios is an important step toward improving one’s quality of life while using | 530 |
| technology wisely and enhancing a productive and healthy lifestyle. | 531 |
|  | 532 |
| **Acknowledgment** | 533 |
| This research was partially supported by The Koret Fund of Digital Living 2030. | 534 |
|  | 535 |
| **References** | 536 |
| Ajzen, Icek. 1991. "The theory of planned behavior". *Organizational Behavior and Human* | 537 |
| *Decision Processes*. 50 (2): 179–211. | 538 |
| Aljomaa, S. S., M. F. Al.Qudah, I. S. Albursan, S. F. Bakhiet, and A. S. Abduljabbar. 2016. | 539 |
| "Smartphone Addiction among University Students in the Light of Some Variables." | 540 |
| *Computers in Human Behavior* 61: 155–164. doi:10.1016/j.chb.2016.03.041. | 541 |
| Al-Samarraie, H., Eldenfria, A., & Dawoud, H. 2017. The impact of personality traits on users’ | 542 |
| information-seeking behavior. *Information Processing & Management*, 53(1), 237-247. | 543 |
| Baruch, Y., and B. C. Holtom. 2008. "Survey Response Rate Levels and Trends in Organizational | 544 |
| Research." *Human Relations* 61 (8): 1139–1160. doi:10.1177/0018726708094863. | 545 |
| Bayoumi, R. R., van der Poel, S. Z., Koert, E., & Boivin, J. 2018. Fertility awareness and quality | 546 |
| of life: assessment and impact of fertility problems and infertility. *Global Reproductive* | 547 |
| *Health*, 3(4), e21. | 548 |

| Boase, J., J. B. Horrigan, B. Wellman, and L. Rainie. 2006. *The Strength of Internet Ties: The* | 549 |
| --- | --- |
| *Internet and E-Mail Aid Users in Maintaining Their Social Networks and Provide Pathways* | 550 |
| *to Help When People Face Big Decisions*. Washington, DC: The Pew Internet and American | 551 |
| Life Project. | 552 |
| Botzer, A., Meyer, J., & Parmet, Y. (2016). Effects of cues in a binary categorization task on | 553 |
| dual-task performance, mental workload, and effort. *Journal of experimental psychology*: | 554 |
| applied, 22, 350. | 555 |
| Brock, D. 1993. "Quality of Life Measures in Health Care and Medical Ethics." In *The Quality of* | 556 |
| *Life*, edited by M. Nussbuam and A. Sen, 95–132. Oxford: Clarendon Press. | 557 |
| Brünken, R., Plass, J. L., & Leutner, D. (2004). Assessment of cognitive load in multimedia | 558 |
| learning with dual-task methodology: Auditory load and modality effects. Instructional | 559 |
| *Science*, 32(1), 115-132. | 560 |
| Buchholz, A., B. Perry, L. B. Weiss, and D. Cooley. 2016. "Smartphone Use and Perceptions | 561 |
| among Medical Students and Practicing Physicians." *Journal of Mobile Technology in* | 562 |
| *Medicine* 5 (1): 27–32. doi:10.7309/jmtm.5.1.5. | 563 |
| Buhrmester, M., T. Kwang, and S. D. Gosling. 2011. "Amazon's Mechanical Turk." *Perspectives* | 564 |
| *on Psychological Science.* 6 (1): 3–5. doi:10.1177/1745691610393980. | 565 |
| Burrows, A. B., Mitchell, V., & Nicolle, C. 2016. “Together: Exploring the Out-of-Box Experience | 566 |
| of Technology for Older Adults.” *International Journal of Mobile Human Computer* | 567 |
| *Interaction*, 8 (2). | 568 |
| Cardinet, J., W. A. N. Tourneur, and L. Allal. 1976. "The Symmetry of Generalizability Theory: | 569 |
| Applications to Educational Measurement." *Journal of Educational Measurement* 13 (2): | 570 |
| 119–135. doi:10.1111/j.1745-3984.1976.tb00003.x. | 571 |
| Chan, M. 2015. "Multimodal Connectedness and Quality of Life: Examining the Influences of | 572 |
| Technology Adoption and Interpersonal Communication on Well-Being across the Life | 573 |
| Span." *Journal of Computer-Mediated Communication* 20 (1): 3–18. doi:10.1111/jcc4.12089. | 574 |
| Chandrasekhar, A., Kim, C. S., Naji, M., Natarajan, K., Hahn, J. O., & Mukkamala, R. (2018). | 575 |
| Smartphone-based blood pressure monitoring via the oscillometric finger-pressing | 576 |
| method. *Science Translational Medicine*, 10(431), eaap8674. | 577 |
| Chesley, N. 2005. "Blurring Boundaries? Linking Technology Use, Spillover, Individual Distress, | 578 |
| and Family Satisfaction." *Journal of Marriage and Family* 67 (5): 1237–1248. | 579 |
| doi:10.1111/j.1741-3737.2005.00213.x. | 580 |
| Cheung, J. H., D. K. Burns, R. R. Sinclair, and M. Sliter. 2017. "Amazon Mechanical Turk in | 581 |
| Organizational Psychology: An Evaluation and Practical Recommendations." *Journal of* | 582 |
| *Business and Psychology* 32 (4): 347–361. doi:10.1007/s10869-016-9458-5. | 583 |

| Clark, L. A., and D. Watson. 1995. "Constructing Validity: Basic Issues in Objective Scale | 584 |
| --- | --- |
| Development." *Psychological Assessment* 7 (3): 309. doi:10.1037/1040-3590.7.3.309. | 585 |
| Collins, A., and E. Koechlin. 2012. "Reasoning, Learning, and Creativity: Frontal Lobe Function | 586 |
| and Human Decision-Making." *PLoS Biology* 10 (3): e1001293. | 587 |
| doi:10.1371/journal.pbio.1001293. | 588 |
| Cotten, S., Cotten, S. R., & Kadylak, T. 2019. The good, the bad, and the ugly: examining outcomes | 589 |
| of technology use among older adults. *Innovation in Aging*, 3(1), S194. | 590 |
| Darcin, A. E., S. Kose, C. O. Noyan, S. Nurmedov, O. Yılmaz, and N. Dilbaz. 2016. "Smartphone | 591 |
| Addiction and Its Relationship with Social Anxiety and Loneliness." *Behavior & Information* | 592 |
| *Technology* 35 (7): 520–525. doi:10.1080/0144929X.2016.1158319. | 593 |
| David, M. E., Roberts, J. A., & Christenson, B. 2018. Too much of a good thing: Investigating the | 594 |
| association between actual smartphone use and individual well-being. *International Journal* | 595 |
| *of Human–Computer Interaction*, 34(3), 265-275. | 596 |
| Derks, D., A. B. Bakker, P. Peters, and P. van Wingerden. 2016. "Work-Related Smartphone Use, | 597 |
| Work–Family Conflict and Family Role Performance: The Role of Segmentation Preference." | 598 |
| *Human Relations* 69 (5): 1045–1068. doi:10.1177/0018726715601890. | 599 |
| Dhillon, Inderjit S., Subramanyam Mallela, and Rahul Kumar. 2003."A divisive information- | 600 |
| theoretic feature clustering algorithm for text classification." *Journal of Machine Learning* | 601 |
| *Research* 3, no. Mar (2003): 1265-1287. | 602 |
| Di, R., & Li, G. 2018. Use of a smartphone medical app improves complications and quality of life | 603 |
| in patients with nasopharyngeal carcinoma who underwent radiotherapy and | 604 |
| chemotherapy. *Medical science monitor: international medical journal of experimental and* | 605 |
| *clinical research*, 24, 6151. | 606 |
| Diener, E., and E. Suh. 1997. "Measuring Quality of Life: Economic, Social, and Subjective | 607 |
| Indicators." *Social Indicators Research* 40 (1): 189–216. doi:10.1023/a:1006859511756. | 608 |
| Dinev, T., & Hu, Q. (2007). The centrality of awareness in the formation of user behavioral | 609 |
| intention toward protective information technologies. *Journal of the Association for* | 610 |
| *Information Systems*, 8(7), 23. | 611 |
| Drago, E. 2015. "The Effect of Technology on Face-to-Face Communication." *Elon Journal of* | 612 |
| *Undergraduate Research in Communications* 6 (1). | 613 |
| Elhai, J. D., Hall, B. J., Levine, J. C., & Dvorak, R. D. 2017. “Types of smartphone access and | 614 |
| relations with problematic smartphone behaviors: The role of content consumption vs. social | 615 |
| smartphone use.” *Cyberpsychology: Journal of Psychosocial Research on Cyberspace*, 11 | 616 |
| (2). | 617 |
| Elhai, J. D., Yang, H., McKay, D., & Asmundson, G. J. (2020). COVID-19 anxiety symptoms | 618 |
| associated with problematic smartphone use severity in Chinese adults. *Journal of Affective* | 619 |
| *Disorders*, 274, 576-582. | 620 |

| Ellis, D. A. (2019). Are smartphones really that bad? Improving the psychological measurement | 621 |
| --- | --- |
| of technology-related behaviors. *Computers in Human Behavior*, 97, 60-66. | 622 |
| Flora, D. B. (2017). The purpose and practice of exploratory and confirmatory factor analysis in | 623 |
| psychological research: Decisions for scale development and validation. Canadian Journal | 624 |
| of Behavioural Science/Revue canadienne des sciences du comportement, 49(2), 78. | 625 |
| Garfin, Dana Rose. "Technology as a coping tool during the coronavirus disease 2019 (COVID‐ | 626 |
| 19) pandemic: Implications and recommendations." *Stress and Health,* 36, no. 4 (2020): | 627 |
| 555-559. | 628 |
| Gilleard, C., I. Jones, and P. Higgs. 2015. "Connectivity in Later Life: The Declining Age Divide | 629 |
| in Mobile Cell Phone Ownership." *Sociological Research Online* 20 (2): 3. | 630 |
| doi:10.5153/sro.3552. | 631 |
| Gist, Marilyn E., and Terence R. Mitchell. 1992. "Self-efficacy: A theoretical analysis of its | 632 |
| determinants and malleability." *Academy of Management Review* 7(2): 183-211. | 633 |
| Glüer, M., & Lohaus, A. 2016. Participation in social network sites: Associations with the quality | 634 |
| of offline and online friendships in German preadolescents and | 635 |
| adolescents. *Cyberpsychology: Journal of Psychosocial Research on Cyberspace*, 10 (2). | 636 |
| Gökçearslan, Ş., F. K. Mumcu, T. Haşlaman, and Y. D. Çevik. 2016. "Modelling Smartphone | 637 |
| Addiction: The Role of Smartphone Access, Self-Regulation, General Self-Efficacy and | 638 |
| Cyberloafing in University Students." *Computers in Human Behavior* 63: 639–649. | 639 |
| doi:10.1016/j.chb.2016.05.091. | 640 |
| Goldberg, A., Ben-Gal, I., Kreitler S. 2016. “The effect of Smartphone Mobility and Connectivity | 641 |
| on Quality of Life”. *Unpublished Thesis.* | 642 |
| Holst, A. (2020, Dec 10). Smartphone users worldwide 2016-2021. Retrieved from | 643 |
| https://www.statista.com/statistics/330695/number-of-smartphone-users-worldwide/ | 644 |
| Hong, F.-Y., S.-I. Chiu, and D.-H. Huang. 2012. "A Model of the Relationship between | 645 |
| Psychological Characteristics, Mobile Phone Addiction and Use of Mobile Phones by | 646 |
| Taiwanese University Female Students." *Computers in Human Behavior* 28 (6): 2152–2159. | 647 |
| doi:10.1016/j.chb.2012.06.020. | 648 |
| Horn, J. L. (1965). A rationale and test for the number of factors in factor analysis. Psychometrika. | 649 |
| 30 (2): 179–185. doi:10.1007/BF02289447. PMID 14306381. S2CID 19663974. | 650 |
| Hu, Li-Tze, and Peter M. Bentler. "Evaluating model fit." (1995). | 651 |
| James, J., and M. Versteeg. 2007. "Mobile Phones in Africa: How Much Do We Really Know?" | 652 |
| *Social Indicators Research* 84 (1): 117–126. doi:10.1007/s11205-006-9079-x. | 653 |
| Johnson, D. 2011. How are Smartphones Being Used?. Accessed 20 November 2012. | 654 |
| http://www.tatango.com/blog/how-are-smartphones-being-used/ | 655 |

| Jovanović, V., M. Joshanloo, D. Đunda, and A. Bakhshi. 2017. "Gender Differences in the | 656 |
| --- | --- |
| Relationship between Domain-Specific and General Life Satisfaction: A Study in Iran and | 657 |
| Serbia." *Applied Research in Quality of Life* 12 (1): 185–204. doi:10.1007/s11482-016-9461- | 658 |
| z. | 659 |
| Kaiser, H. F. (1960). The Application of Electronic Computers to Factor Analysis. *Educational* | 660 |
| *and Psychological Measurement*. 20 (1): 141–151. doi:10.1177/001316446002000116. | 661 |
| Katinka, B., and K. Paul. 2003. "Introduction: Trust within Organisations.", Personnel Review 32 | 662 |
| (5): 543–555. doi:10.1108/00483480310488324. | 663 |
| Kaufman, Leonard, and Peter J. Rousseeuw. 2009. Finding groups in data: an introduction to | 664 |
| cluster analysis. Vol. 344. John Wiley & Sons. | 665 |
| Kendall, Maurice G. 1938. "A new measure of rank correlation." *Biometrika* 30 (1/2): 81-93. | 666 |
| Koch, C. G., Khandwala, F., Cywinski, J. B., Ishwaran, H., Estafanous, F. G., Loop, F. D., & | 667 |
| Blackstone, E. H. 2004. Health-related quality of life after coronary artery bypass grafting: a | 668 |
| gender analysis using the Duke Activity Status Index. *The Journal of Thoracic and* | 669 |
| *Cardiovascular Surgery*, 128(2), 284-295. | 670 |
| Kreitler, S., and M. M. Kreitler. 2006. "Multidimensional Quality of Life: A New Measure of | 671 |
| Quality of Life in Adults." *Social Indicators Research* 76 (1): 5–33. doi:10.1007/s11205-005- | 672 |
| 4854-7. | 673 |
| Kuhlmann, T. 2018. Optimizing measurement in Internet-based research: Response scales and | 674 |
| sensor data (Doctoral dissertation). | 675 |
| Land, K. C. 1996. "Social Indicators and the Quality of Life: Where Do We Stand in the Mid- | 676 |
| 1990s?" *SINET* 45: 5–8. | 677 |
| Lee, Y.-K., C.-T. Chang, Z.-H. Cheng, and Y. Lin. 2016. "Helpful-Stressful Cycle? Psychological | 678 |
| Links between Type of Mobile Phone User and Stress." *Behavior & Information Technology* | 679 |
| 35 (1): 75–86. doi:10.1080/0144929X.2015.1055800. | 680 |
| Lee, Y.-K., C.-T. Chang, Y. Lin, and Z.-H. Cheng. 2014. "The Dark Side of Smartphone Access: | 681 |
| Psychological Traits, Compulsive Behavior and Technostress." *Computers in Human* | 682 |
| *Behavior* 31: 373–383. doi:10.1016/j.chb.2013.10.047. | 683 |
| Levine, E. G., G. J. Yoo, and C. Aviv. 2017. "Predictors of Quality of Life among Ethnically | 684 |
| Diverse Breast Cancer Survivors." *Applied Research in Quality of Life* 12 (1): 1–16. | 685 |
| doi:10.1007/s11482-016-9447-x. | 686 |
| Ley, B., C. Ogonowski, J. Hess, T. Reichling, L. Wan, and V. Wulf. 2014. "Impacts of New | 687 |
| Technologies on Media Access and Social Behavior in Domestic Environments." *Behavior &* | 688 |
| *Information Technology* 33 (8): 815–828. doi:10.1080/0144929X.2013.832383. | 689 |
| Lilly, J. D., Duffy, J. A., & Virick, M. (2006). A gender-sensitive study of McClelland's needs, | 690 |
| stress, and turnover intent with work-family conflict. *Women in Management Review*. | 691 |

| Ling, R., Kaufmann, M. 2005. “The Mobile Connection: The Cell Phone’s Impact on Society.” | 692 |
| --- | --- |
| Book Review by Love S., *International Journal of Technology and Human Interaction* 1 (4): | 693 |
| 101-104. | 694 |
| Lior, R., & Maimon, O. (2005). Clustering methods. *Data mining and knowledge discovery* | 695 |
| *handbook*, 321-352. | 696 |
| Litchfield, S. 2010. Defining the Smartphone. Accessed June 1, 2015, | 697 |
| http://www.allaboutsymbian.com/features/item/Defining_the_Smartphone.php. | 698 |
| Longstreet, P., & Brooks, S. 2017. Life satisfaction: A key to managing internet & social media | 699 |
| addiction. *Technology in Society*, 50, 73-77. | 700 |
| Lord, C., M. Hazas, A. K. Clear, O. Bates, R. Whittam, J. Morley, and A. Friday. 2015. "Demand | 701 |
| in My Pocket: Mobile Devices and the Data Connectivity Marshalled in Support of Everyday | 702 |
| Practice." Paper presented at the Proceedings of the 33rd Annual ACM Conference on Human | 703 |
| Factors in Computing Systems, Seoul, 2729–2738. | 704 |
| Maimon, O., & Rokach, L. (2005). Decomposition Methodology for Knowledge Discovery and | 705 |
| Data Mining. In O. Maimon, & L. Rokach (Eds.), *Data Mining and Knowledge Discovery* | 706 |
| *Handbook* (pp. 981-1003). Boston, MA: Springer US. doi:10.1007/0-387-25465-X_46 | 707 |
| Markovic, G., M. S. Louise, and A. Bartfai. 2017. "The effect of sampling bias on generalizability | 708 |
| in intervention trials after brain injury." *Brain injury* 31 (1): 9-15. | 709 |
| Mart, M., Thompson, J., Ely, E. W., Pandharipande, P., Patel, M., Wilson, J. E. and Brummel, N. | 710 |
| E. 2019. Relationship of Level of Consciousness in Critical Illness with Disability and Quality | 711 |
| of Life. In B104. Critical care: catch-22-beyond the ventilator: the challenges of delirium, icu- | 712 |
| acquired weakness, and rehabilitation (pp. a4106-a4106). *American Thoracic Society*. | 713 |
| McDaniel, B. T., and S. M. Coyne. 2016. “Technoference”: The Interference of Technology in | 714 |
| Couple Relationships and Implications for Women’s Personal and Relational Well-Being." | 715 |
| *Psychology of Popular Media Culture* 5 (1): 85. doi:10.1037/ppm0000065. | 716 |
| Middleton, C., R. Scheepers, and V. K. Tuunainen. 2014. "When Mobile Is the Norm: Researching | 717 |
| Mobile Information Systems and Mobility as Post-Adoption Phenomena." *European Journal* | 718 |
| *of Information Systems* 23 (5): 503–512. doi:10.1057/ejis.2014.21. | 719 |
| Mieczakowski, A., T. Goldhaber, and P. Clarkson. 2011. *Culture, Communication and Change:* | 720 |
| *Summary of an Investigation of the Use and Impact of Modern Media and Technology in Our* | 721 |
| *Lives*. Cambridge: Cambridge University Press. | 722 |
| Misra, S., and D. Stokols. 2012. "Psychological and Health Outcomes of Perceived Information | 723 |
| Overload." *Environment and Behavior* 44 (6): 737–759. doi:10.1177/0013916511404408. | 724 |
| Misra, S., L. Cheng, J. Genevie, and M. Yuan. 2016. "The Iphone Effect." *Environment and* | 725 |
| *Behavior* 48 (2): 275–298. doi:10.1177/0013916514539755. | 726 |
| Murtagh, Fionn. 1983. "A survey of recent advances in hierarchical clustering algorithms." *The* | 727 |
| *Computer Journal* 26 (4): 354-359. | 728 |

| Newzoo, Top Countries by Smartphone Penetration & Users, Retrieved May 8, 2017, from, | 729 |
| --- | --- |
| 2017,[https://newzoo.com/insights/rankings/top-50-countriesby-s](https://newzoo.com/insights/rankings/top-50-countriesby-)martphone-penetration-and- | 730 |
| users/. | 731 |
| Noll, H.-H. 2004. "Social Indicators and Quality of Life Research: Background, Achievements and | 732 |
| Current Trends." In *Advances in Sociological Knowledge: Over Half a Century*, edited by N. | 733 |
| Genov, 151–181. Wiesbaden: VS Verlag für Sozialwissenschaften. | 734 |
| Paas, F., Tuovinen, J. E., Tabbers, H., & Van Gerven, P. W. (2003). Cognitive load measurement | 735 |
| as a means to advance cognitive load theory. *Educational psychologist*, 38(1), 63-71. | 736 |
| Panagiotidi, M., & Overton, P. (2018). The relationship between internet addiction, attention | 737 |
| deficit hyperactivity symptoms and online activities in adults. *Comprehensive psychiatry*, 87, | 738 |
| 7-11. | 739 |
| Park, N., and H. Lee. 2012. "Social Implications of Smartphone Use: Korean College Students' | 740 |
| Smartphone Use and Psychological Well-Being." *Cyberpsychology Behavior and Social* | 741 |
| *Networking* 15 (9): 491–497. doi:10.1089/cyber.2011.0580. | 742 |
| Park, N., S. Lee, and J. E. Chung. 2016. "Uses of Cellphone Texting: An Integration of | 743 |
| Motivations, Access Patterns, and Psychological Outcomes." *Computers in Human Behavior* | 744 |
| 62: 712–719. doi:10.1016/j.chb.2016.04.041. | 745 |
| Pearson, A. L., E. Mack, and J. Namanya. 2017. "Mobile Phones and Mental Well-Being: Initial | 746 |
| Evidence Suggesting the Importance of Staying Connected to Family in Rural, Remote | 747 |
| Communities in Uganda." PloS one 12 (1). e0169819. | 748 |
| Peterson, R. A. (2000). A meta-analysis of variance accounted for and factor loadings in | 749 |
| exploratory factor analysis. Marketing letters, 11(3), 261-275. | 750 |
| Quan-Haase, A., and J. L. Collins. 2008. "‘I'm There, but I Might Not Want to Talk to You’." | 751 |
| Information, Communication & Society 11 (4): 526–543. doi:10.1080/13691180801999043. | 752 |
| Rai, S., S. Saroshe, A. K. Khatri, S. Sirohi, and S. Dixit. 2017. "A Cross Sectional Study to Assess | 753 |
| the Effects of Excessive Use of Smartphones among Professional College Going Students." | 754 |
| *International Journal of Community Medicine and Public Health* 3 (3): 758–763. | 755 |
| doi:10.18203/2394-6040.ijcmph20160647. | 756 |
| Rainie, L., and B. Wellman. 2012. *Networked: The New Social Operating System*. Cambridge, | 757 |
| MA: MIT Press. | 758 |
| Rosseel, Y. (2012). lavaan: An R Package for Structural Equation Modelling. *Journal of* | 759 |
| *Statistical Software*, 48(2), 1-36. Retrieved from http://www.jstatsoft.org/v48/i02/ | 760 |
| Rotondi, V., Stanca, L., & Tomasuolo, M. (2017). Connecting alone: Smartphone use, quality of | 761 |
| social interactions and well-being. *Journal of Economic Psychology*, 63, 17-26. | 762 |
| Rouse, M. 2007. Cellular Phone (mobile telephone). Accessed June 1, 2015, | 763 |
| http://searchmobilecomputing.techtarget.com/definition/cellular-telephone | 764 |

| Salehan, M., and A. Negahban. 2013. "Social Networking on Smartphones: When Mobile Phones | 765 |
| --- | --- |
| Become Addictive." *Computers in Human Behavior* 29 (6): 2632–2639. | 766 |
| doi:10.1016/j.chb.2013.07.003. | 767 |
| Samaha, M., and N. S. Hawi. 2016. "Relationships among Smartphone Addiction, Stress, | 768 |
| Academic Performance, and Satisfaction with Life." *Computers in Human Behavior* 57: 321– | 769 |
| 325. doi:10.1016/j.chb.2015.12.045. | 770 |
| Sela, A. (n.d.). Github. Retrieved from Article’s GitHub repository: | 771 |
| https://github.com/alonsela2015/Smartphones_QualityOfLife | 772 |
| Setz, C., Arnrich, B., Schumm, J., La Marca, R., Tröster, G., & Ehlert, U. (2009). Discriminating | 773 |
| stress from cognitive load using a wearable EDA device. *IEEE Transactions on information* | 774 |
| *technology in biomedicine*, 14(2), 410-417. | 775 |
| Sirgy, M. J., and D.-J. Lee. 2015. "Work-Life Balance: A Quality-of-Life Model." *Applied* | 776 |
| *Research in Quality of Life* 11 (4): 1059–1082. doi:10.1007/s11482-015-9419-6. | 777 |
| Škařupová, K., K. Ólafsson, and L. Blinka. 2016. "The Effect of Smartphone Use on Trends in | 778 |
| European Adolescents’ Excessive Internet Use." *Behavior & Information Technology* 35 (1): | 779 |
| 68–74. doi:10.1080/0144929X.2015.1114144. | 780 |
| Smith, A. 2012. 46% of American Adults Are Smartphone Owners Phones within, Pew Research | 781 |
| Center’s Internet& American Life Project. Accesses June 1, 2015, | 782 |
| [http://pewinternet.org/Reports/2012/Smartphone-Update-2012.aspx.](http://pewinternet.org/Reports/2012/Smartphone-Update-2012.aspx) | 783 |
| Suhr, Diana D., Exploratory or confirmatory factor analysis? (2006): 66-76. | 784 |
| Sweller, J. (2011). Cognitive load theory*. In Psychology of learning and motivation* (Vol. 55, pp. | 785 |
| 37-76). Academic Press. | 786 |
| Szameitat, A. J., Schubert, T., Müller, K., & Von Cramon, D. Y. (2002). Localization of executive | 787 |
| functions in dual-task performance with fMRI. *Journal of cognitive neuroscience,* 14(8), | 788 |
| 1184-1199. | 789 |
| Techatassanasoontorn, A., & Tanvisuth, A. 2010. IS Use and Quality of Life: A Conceptualization | 790 |
| and Empirical Investigation. *AIS Transactions on Human-Computer Interaction, 2*(2), 26-54. | 791 |
| Retrieved from https://aisel.aisnet.org/thci/vol2/iss2/3 | 792 |
| Thomée, S., A. Härenstam, and M. Hagberg. 2011. "Mobile Phone Use and Stress, Sleep | 793 |
| Disturbances, and Symptoms of Depression among Young Adults-A Prospective Cohort | 794 |
| Study." *BMC Public Health* 11 (1): 66. doi:10.1186/1471-2458-11-66. | 795 |
| Thompson, B. (2004). Exploratory and confirmatory factor analysis. *American Psychological* | 796 |
| *Association.* | 797 |
| Turkle, S. 2011. *Life on the Screen*. New York, NY: Simon & Schuster. | 798 |
| Uysal, M., M. J. Sirgy, E. Woo, and H. Kim. 2016. "Quality of Life (QOL) and Well-Being | 799 |
| Research in Tourism." *Tourism Management* 53: 244–261. | 800 |
| doi:10.1016/j.tourman.2015.07.013. | 801 |

| Vance, Ashlee (2011-04-14). ["This Tech Bubble Is Different".](https://www.bloomberg.com/news/articles/2011-04-14/this-tech-bubble-is-different) Bloomberg.com. Retrieved 2017- | 802 |
| --- | --- |
| 08-29. | 803 |
| Ventegodt, S., Andersen, N. J., & Merrick, J. 2003. Quality of Life Philosophy IV. The Brain and | 804 |
| Consciousness. *The Scientific World Journal*, 3, 1199-1209. | 805 |
| Virick, M., DaSilva, N., & Arrington, K. (2010). Moderators of the curvilinear relation between | 806 |
| extent of telecommuting and job and life satisfaction: the role of performance outcome | 807 |
| orientation and worker type. *Human relations*, 63, 137-154. | 808 |
| Wajcman, J., M. Bittman, P. Jones, L. Johnstone, and J. Brown. 2008. *The Impact of the Mobile* | 809 |
| *Phone on Work/Life Balance -Australian Research Council Linkage Project Final Survey* | 810 |
| *Report*. Australian National University: Australian Mobile Telecommunications Association. | 811 |
| Wajcman, J., M. Bittman, and J. E. Brown. 2008. "Families without Borders: Mobile Phones, | 812 |
| Connectedness and Work-Home Divisions." *Sociology* 42 (4): 635–652. | 813 |
| doi:10.1177/0038038508091620. | 814 |
| Wang, J.-L., H.-Z. Wang, J. Gaskin, and L.-H. Wang. 2015. "The Role of Stress and Motivation | 815 |
| in Problematic Smartphone Use among College Students." *Computers in Human Behavior* | 816 |
| 53: 181–188. doi:10.1016/j.chb.2015.07.005. | 817 |
| Yun, H., W. J. Kettinger, and C. C. Lee. 2012. "A New Open Door: The Smartphone's Impact on | 818 |
| Work-to-Life Conflict, Stress, and Resistance." *International Journal of Electronic* | 819 |
| *Commerce* 16 (4): 121–152. doi:10.2753/JEC1086-4415160405. | 820 |
| Zilka, G. C. 2018. “Аlways with them: smartphone use by children, adolescents, and young | 821 |
| adults—characteristics, habits of use, sharing, and satisfaction of needs.” *Universal Access in* | 822 |
| *the Information Society*, 1-11. | 823 |
| Zwick, W. R., & Velicer, W. F. (1986). Comparison of five rules for determining the number of | 824 |
| components to retain. *Psychological Bulletin*. 99 (3): 432–442. doi:10.1037//0033- | 825 |
| 2909.99.3.432. | 826 |
| **SUPPLEMENTARY MATERIAL** | 827 |
| **1. CFA Analysis & SEM models** | 828 |
| MODEL 1 - QOL to Usability | 829 |
| 'competence =~ y2 + y12 + y15 + y16 + y17 + y18 | 830 |
| functioning =~ y3 +y4+y5+y6+y9+y10+y11+y13+y14+ y21 | 831 |
| positive_feeling =~ y1 + y7 + y8 + y19 + y20 + y22 | 832 |
| unaware =~ x4+x3+x5+x6+x12+x11+x10 | 833 |
| aware =~ x2+x7+x8+x9 | 834 |
| y13 ~~ y14 | 835 |
| y4 ~~ y3 | 836 |
| y12 ~~ y15 | 837 |
| competence ~ unaware + aware | 838 |

| functioning ~ unaware + aware | 839 |
| --- | --- |
| positive_feeling ~ unaware + aware' | 840 |
|  | 841 |
| MODEL 2: Usability to QOL | 842 |
| full.model2 <- 'competence =~ y2 + y12 + y15 + y16 + y17 + y18 | 843 |
| functioning =~ y3 +y4+y5+y6+y9+y10+y11+y13+y14+ y21 | 844 |
| positive_feeling =~ y1 + y7 + y8 + y19 + y20 + y22 | 845 |
| unaware =~ x4+x3+x5+x6+x12+x11+x10 | 846 |
| aware =~ x2+x7+x8+x9 | 847 |
| y13 ~~ y14 | 848 |
| y4 ~~ y3 | 849 |
| y12 ~~ y15 | 850 |
| unaware ~ competence + functioning + positive_feeling | 851 |
| aware ~ competence + functioning + positive_feeling' | 852 |
|  | 853 |
|  | 854 |
|  | 855 |
|  | 856 |
|  | 857 |
|  | 858 |
|  | 859 |
|  | 860 |
|  | 861 |

# 1. CFA results 862

 863

864

865

# 2. QOL questionnaire. 866

These questions, copied from Kreitler and Kreitler (2006), measure the levels of QOL through	867

the positive feelings, competence and functioning subfactors.	868

|  | There is a lot | To some extent | Not so much | None at all |
| --- | --- | --- | --- | --- |
| A general sense of good physical health |  |  |  |  |
| Pain |  |  |  |  |
| Work (outside the home or in the home, including caring for the household) |  |  |  |  |
| Activity (outside the home or inside the home) |  |  |  |  |
| Strength and ability to perform the activities of eating, sleeping, etc. |  |  |  |  |
| Strength and ability to perform activities in the family (as a partner, parent, sibling, son/daughter) |  |  |  |  |
| Intimate relations with your partner |  |  |  |  |
| Relations with your friends, acquaintances, relatives |  |  |  |  |
| Taking care of yourself and your external appearance |  |  |  |  |
| Strength and ability to cope with the tasks of your everyday life |  |  |  |  |
| Independence in functioning and activities in your daily life |  |  |  |  |
| Not experiencing a sense of a lack of control over situations, feeling that you cannot determine anything that happens |  |  |  |  |
| Ability to concentrate on the task you are performing |  |  |  |  |
| Ability to think, solve problems |  |  |  |  |
| Sense of uncertainty, lack of clarity |  |  |  |  |
| Anxiety, fear |  |  |  |  |
| Depression, sadness |  |  |  |  |
| Tension, restlessness |  |  |  |  |
| Hope |  |  |  |  |
| Pleasure |  |  |  |  |
| Motivation to make an effort and continue doing things |  |  |  |  |
| Satisfaction with life in general |  |  |  |  |

869
