# Supplementary material for: Smartphone use behavior and quality of life: What is the role of awareness?
Source: PLoS One. 2022 Mar 11;17(3):e0260637. doi: 10.1371/journal.pone.0260637 (PMC8916658; doi:10.1371/journal.pone.0260637)
Supplement: Attachment — Submitted filename: Response to Reviewers_v281021.docx [file pone.0260637.s003.docx]

Dear Dr. Bilan

Thank you for inviting us to resubmit a revised version of the manuscript, “*Smartphone use behavior and quality of life: what is the role of awareness?*”, number PONE-D-21-20673.

The manuscript has been revised according to the comments of the reviewers.

In this letter we address each of the comment of the reviewers, as well as the comments of the editor. For ease of reading, we first copied the reviewers` comments followed by our response.

We hope you will find the revised manuscript adequate for publication in PLOS ONE.

Looking forward to hearing from you,

Alon Sela, on behalf of the authors.

__________________________________________________________________________________

**Journal requirements and general comments:**

1. We followed the journal guidelines to ensure the manuscript meets PLOS ONE`s style requirements.

**Response:** We followed the guidelines to ensure PLOS ONE style

2. Please remove any funding-related text from the manuscript and let us know how you would like to update your Funding Statement. Currently, your Funding Statement reads as follows:

**Response:** Thank you for your comment. We removed the funding statement from the acknowledgement and only mention it in the PLOS system.

3. Your ethics statement should only appear in the Methods section of your manuscript. If your ethics statement is written in any section besides the Methods, please delete it from any other section.

**Response:** We verified that the ethics statement only appears in the Methods section. We moved the ethics statement to section Methods section, directly after the data collection section (named Data) since we believe that at this location it is more fluent to the reader.

5. We note that you have referenced (Goldberg, A., Ben-Gal, I., Kreitler S. 2016. “The effect of Smartphone Mobility and Connectivity 641 on Quality of Life”) which has currently not yet been accepted for publication. Please remove this from your References and amend this to state in the body of your manuscript: (Goldberg, A., Ben-Gal, I., Kreitler S. 2016. “The effect of Smartphone Mobility and Connectivity 641 on Quality of Life”. Unpublished Thesis) as detailed online in our guide for authors

<http://journals.plos.org/plosone/s/submission-guidelines#loc-reference-style> "

**Response:** We removed this unpublished reference from the article since we could not obtain a corresponding DOI number as required.

6. Other benefits of using smartphones and ICT should be considered. For example, ICT and Economic Growth: Links and Possibilities of Engaging. <https://doi.org/10.13165/IE-19-13-1-07>

**Response:** Thank you for your comment. We addressed this issue and added the mentioned reference. Additionally, we elaborated on the additional benefits of smartphone use.

**Reviewer #1 comments:**

We would like to thank the reviewer for his valuable comments. We have thoroughly studied the reviewer’s comments and modified the manuscript accordingly.

1. 57) It is worth mentioning that QOL research spans the fields not only of psychology, philosophy, social psychology, but also economics. For example, a noteworthy QOL analysis was performed in DOI: 10.1080 / 1331677X.2021.1956361

**Response:** We added a reference to economics and mention some additional recent studies: “*QOL research spans the fields of psychology (Land, 1996), philosophy (Brock, 1993), social psychology (Noll, 2004) and economics (Tvaronaviciene, 2021).” Furthermore,* *“…a recent study (Tvaronaviciene, 2021) has found that QOL measurement for young adults should include the economic factors.”*

2. 143, 179) should improve the description of the methodology for determining the essence of such smartphone usability latent variables as ‘aware’ and ‘unaware’ use behavior modes. In our opinion, it can be learned starting from line 323 of table 1.

**Response:** We appreciate this comment. We added the paragraph below to better explain the research methodological approach. Additionally, we provide the location in the article where it is explained:

*We followed conventional data mining and machine learning methodologies and borrowed methods from disciplines presented in (Suhr, 2006), (Zwick & Velicer, 1986), (Peterson, 2000).* *According to this analysis path, the study first explores the data through known statistical tools and then build a learning model to better explain the results that were emerged from the data analysis stage. Therefore, we first conducted a preliminary dimensionality reduction procedure, where each distinct question in the questionnaire was considered as a dimension, in order to find the most informative dimensions in the questionnaire. Note that in such an analysis, the higher dimensions are conceptually equivalent to Latent Variables that are constructed of numerous questions vectors.*

*Following this preliminary analysis, we found that it is best to separate the QOL questions into three main subcomponents (i.e., the latent variables) and the smartphone usage questions into two latent variables. The full description of the procedure that was used to find the number of latent variables as well as how to determine the questions that appear in each latent variable are now presented in Fig 2, Fig 3, Table 1 and Table 2 in the results section below.*

3. 185, 195, 219) should improve the description of the methodology for determining the essence of such latent QOL variables as 'competence,' 'functioning,' and 'positive feeling.' In our opinion, it can be found starting from line 325 table 2.

**Response:** Thank you for your comment. We added the following paragraph to better describe the means by which each latent variable was named.

*In accordance with the QOL subcomponents developed by Kreitler and Kreitler (2006), we named the three latent QOL variables - 'competence,' 'functioning,' and 'positive feeling' as in the original QOL scale. To better grasp the items’ meaning, one can look at Table 2, where the correlation coefficients between the items and the three QOL latent variables are presented. Generally, positive feelings is a cluster where people experience feelings such as ‘hope’, ‘pleasure’, ‘satisfaction’, ‘good relations with others’ and ‘good health’. Competence includes feelings such as ‘sense of control over life`s situations’, ‘clarity’, ‘lack of anxiety’, ‘lack of depression’ or ‘lack of tension’. Functioning includes feeling of ‘active lifestyle’, ‘functioning in work’, and feelings of strength* *in performing basic daily activities, such as sleeping, eating, and overall,* *a good ability to easily perform basic daily tasks.*

4. Tables 1 and 2 are mentioned in the text in lines 93, 166, although given in lines 323 and 325.

**Response:** We appreciate the comment but nevertheless, kept the structure in which we first generally describe the latent variables, and only then in the tables, drill down into more detailed explanation. We believe that this format makes the reading easier to comprehend.

5. 257) In the Results section, you should leave the information in paragraph 3.4, which clearly corresponds to the title of the article "Smartphone Use Behavior and Quality of Life: What Is the Role of Awareness?"

**Response:** We thank and appreciate your comment. Following this comment we added a clarification at the beginning of the paragraph, stating:

*We used the Structural Equation Modeling (SEM) method to examine the complex sets of interactions between the use behavior variables and the quality-of-life variables.*

6. 115) the article does not substantiate the representativeness of the sample of 215 respondents.

**Response:** The following text was added to the article to clarify the representativeness of the sample (4 in the SI section).

*The sample size was based on a pre-experiment analysis of the first 50 respondents**, in which we estimated the normalized QOL standard deviation SD=0.22. Based on this estimate, the sample size was defined (using a Z-score=1.96 with 95% confidence level and 5% precision), to be* $\frac{{1.96}^{2}*0.22*(1-0.22)}{0.0025}=263$ *respondents.* *As the data collection from the questionnaire progressed, we updated the standard deviation estimations to 0.194. These corresponded with a sample size of 234 individuals. Following this stage, 19 questionnaires were excluded due to poor data quality, resulting in 215 full usable questionnaires.* *This sample size fits a precision level of 0.052, which is slightly higher that the desired 0.05 precision level but based on higher quality and more reliable questionnaires. Furthermore, the SD of the Aware/Unaware latent variables was slightly smaller (0.19 vs 0.17), supporting our decision.*

7. 323, 325) the name of tables 1 and 2 should be changed, because, as we understand here, the correlation coefficients are given.

**Response:** We appreciate this comment. The tables’ names were changed to better correspond to their content.

Table 1. Correlation coefficient between each item and the two smartphone use latent variables

Table 2. Correlation coefficient between each item and the three QOL latent variables

8. 323, 325) the names of the graphs in the header are not fully displayed, it is desirable to place the words in a different order so that they can be read by turning the sheet clockwise

**Response:** The tables names were changed and the pages where the tables appear were adjusted to Landscape mode for easier reading.

9. 291) Figure 3 is mentioned in line 291, and is given in line 316; the signature of the figure is not on the same page with the figure; to make Figure 3 more compact, variable signatures can be matched to Tables 1 and 2: x1-x12, y1-y22.

**Response:** Thank you for your comment. While noting the variable as x1-x12, y1-y22 can make the image more compact, it will also make it harder for the reader to grasp the meaning of each variable (since one would need to see a translation map between x1-x12, y1-y22 and the real questions items). Thus, we believe that keeping the existing format may ease the reader`s understanding regarding the correlations between the various questions.

10. 377) the name of table 3 is detached from the body of the table

**Response:** We corrected by space adjustments.

11. 368-369) there are errors in the sentence

**Response:** We removed the unnecessary punctuation point that was in the sentence and create the error. The sentence changed to the original meaning of the sentence. “*…; however, it could be that it is the QOL that affects smartphone use and not the opposite. Fig 4 (right)…*”

**Reviewer #2 Comments:**

We would like to thank the reviewer for his valuable comments. We have thoroughly studied the reviewer’s comments and modified the manuscript accordingly.

1. Other benefits of using smartphones and ICT should be considered.

**Response:** We appreciate this helpful comment. We added other benefits of using smartphones, specifically related to the economic value as a tool enhancing economic development as it was mentioned in the article referenced.

“*Smartphones are nowadays an entry point to the Internet, a portable computer that speeds up the transfer of knowledge, enhance e-commerce, and as such are important for economic growth. This is also why their use rates are mentioned in the Global Competitive Index (Bilan et al 2019)*.”

Consequently, we added the ref. “Bilan, Y., Mishchuk, H., Samoliuk, N., & Grishnova, O. (2019). ICT and economic growth: Links and possibilities of engaging*. Intellectual Economics, 13(1), 93-104*.‏” into the bibliographic list.

2. It is necessary to describe how the sample was formed and assess the reliability of the information obtained by the questionnaire.

**Response**: Following this remark, we added a new section, supported by additional analyses that were added to Section 3 in the SI (Supplementary Information) section. Our analyses include the tables detailing the Alpha Cronbach coefficient, the Interclass Correlation Coefficient analysis as well as additional analyses which addresses this important remark.

*We tested the reliability by using both the Cronbach's Alpha and the McDonald's Omega estimators. The QOL Cronbach's Alpha of the Competence, Functioning and Positive Feelings latent variables were 0.86, 0.92, 0.82 respectively (see Table SI-4 below). For the Aware component is slightly lower than 0.6, which is low, but nevertheless, it is considered as not unacceptable (eg, George & Mallery, 2003). Also, as can be seen from the regression coefficient in Table SI-3, the Aware and the Unaware coefficients are in inverse directions for all 3 usability variables, i.e. (-0.398, 0.319), (-0.333, 0.223), (-0.408, 0.5). Thus, these factors, although possibly not clustered around a single clear latent variable named Aware usability, are different from the Unaware usability variables.*

| *Table SI-4* |  |  | |  |  |  |
| --- | --- | --- | --- | --- | --- | --- |
| *Reliability by Cronbach Alpha and McDonald`s Omega* | *Competence* | *Functioning* | *Positive feeling* | | *Unaware* | *Aware* |
| *Cronbach Alpha* | *0.859* | *0.902* | *0.8217* | | *0.77* | *0.592* |
| *McDonald's Omega* | *0.862* | *0.904* | *0.823* | | *0.774* | *0.594* |

*Also, we tried to remove from the Aware latent variable X1, X2, X8, X9, but this did not result in a better reliability for the Aware construct. Finally, we performed an interclass correlation coefficient analysis, (see Table SI-5 below). One can see that the Cronbach Alpha for the Aware construct is within the 95% confidence intervals.*

***Table Si-5 - Interclass Correlation Coefficient***

|  |  | *95% Confidence interval* | |  |  |
| --- | --- | --- | --- | --- | --- |
|  | *Interclass Correlation* | *Lower Bound* | *Upper Bound* | *Value* | *Significance* |
| *Single Measure* | *0.225* | *0.166* | *0.292* | *2.45* | *0* |
| *Average Measure* | *0.592* | *0.498* | *0.673* | *2.45* | *0* |

3. Some literary sources are outdated.

**Response:** Thank you for this important comment. We added and validated the literary sources. We removed outdated literary sources. The reference in the clean version are according to the required style [1], [2],…, but we did not change the style in the marked version for ease of the reviewers follow-up.

We thank again the editor and the reviewers for their insightful and important comments. We believe that the modifications following these comments significantly improve the manuscript.

We hope that in the attached revised version, in which we addressed all the reviewers’ comments, is ready for publication in PLOS 1.

Sincerely,

Dr. Alon Sela, on behalf of the authors.

[Alonse2012@gmail.com](mailto:Alonse2012@gmail.com)
